# Genome-wide analysis in *Plasmodium falciparum* reveals early and late phases of RNA polymerase II occupancy during the infectious cycle

**DOI:** 10.1186/1471-2164-15-959

**Published:** 2014-11-06

**Authors:** Ragini Rai, Lei Zhu, Haifen Chen, Archana Patkar Gupta, Siu Kwan Sze, Jie Zheng, Christiane Ruedl, Zbynek Bozdech, Mark Featherstone

**Affiliations:** School of Biological Sciences, Nanyang Technological University, 60 Nanyang Drive, Singapore, 637551 Singapore; School of Computer Engineering, Nanyang Technological University, Block N4 #02a-32 Nanyang Avenue, Singapore, 639798 Singapore

## Abstract

**Background:**

Over the course of its intraerythrocytic developmental cycle (IDC), the malaria parasite *Plasmodium falciparum* tightly orchestrates the rise and fall of transcript levels for hundreds of genes. Considerable debate has focused on the relative importance of transcriptional versus post-transcriptional processes in the regulation of transcript levels. Enzymatically active forms of RNAPII in other organisms have been associated with phosphorylation on the serines at positions 2 and 5 of the heptad repeats within the C-terminal domain (CTD) of RNAPII. We reasoned that insight into the contribution of transcriptional mechanisms to gene expression in *P. falciparum* could be obtained by comparing the presence of enzymatically active forms of RNAPII at multiple genes with the abundance of their associated transcripts.

**Results:**

We exploited the phosphorylation state of the CTD to detect enzymatically active forms of RNAPII at most *P. falciparum* genes across the IDC. We raised highly specific monoclonal antibodies against three forms of the parasite CTD, namely unphosphorylated, Ser5-P and Ser2/5-P, and used these in ChIP-on-chip type experiments to map the genome-wide occupancy of RNAPII. Our data reveal that the IDC is divided into early and late phases of RNAPII occupancy evident from simple bi-phasic RNAPII binding profiles. By comparison to mRNA abundance, we identified sub-sets of genes with high occupancy by enzymatically active forms of RNAPII and relatively low transcript levels and *vice versa*. We further show that the presence of active and repressive histone modifications correlates with RNAPII occupancy over the IDC.

**Conclusions:**

The simple early/late occupancy by RNAPII cannot account for the complex dynamics of mRNA accumulation over the IDC, suggesting a major role for mechanisms acting downstream of RNAPII occupancy in the control of gene expression in this parasite.

**Electronic supplementary material:**

The online version of this article (doi:10.1186/1471-2164-15-959) contains supplementary material, which is available to authorized users.

## Background

The malaria parasite *Plasmodium falciparum* undergoes a 48 h life cycle from the moment of red blood cell invasion through to the production and release of mature progeny. In the course of this intraerythrocytic developmental cycle (IDC), the mRNA level for many genes rises and falls once at a point that correlates with the time its protein product is needed. Such results have led to the proposal of a “just in time” model of plasmodial gene expression in which mRNAs accumulate just as their products are required during the IDC [1] Many factors affect mRNA levels, including transcriptional initiation, transcriptional elongation, mRNA processing, mRNA export, and mRNA stability. While control of *Plasmodium* gene expression at the level of transcription has been demonstrated [2–5], several recent studies provide evidence that post-transcriptional mechanisms must play a major role as well. For example, *P. falciparum* shows a widespread chromatin opening and histone H2A.Z recruitment in the intergenic regions throughout the IDC. Although this modification has been associated with actively transcribed chromatin in other species, in *Plasmodium falciparum* this histone variant is also recruited early to genes whose transcripts do not appear until much later [6–8]. A similar disconnect is seen with component of the basal transcriptional machinery, TBP and TFIIE, which are broadly recruited to *Plasmodium* genes regardless of corresponding transcript abundance [9]. Moreover, nuclear run-ons correlated active transcription of some selected genes with the levels of their transcripts across the IDC. While some loci showed clear positive correlation between transcriptional activity and mRNA abundance, others revealed striking discrepancies strongly indicative of post-transcriptional regulation [10] and consistent with similar discordances seen in humans [11]. Additionally, transcript stability has been demonstrated to vary by an average of six fold between ring and schizont stages, and is correlated with a progressive loss of RNA degrading enzymes [12]. Last, parasites deficient in the post-transcriptional regulator CAF1 display major shifts in peaks of mRNA accumulation [13]. Such findings are consistent with major post-transcriptional control during the IDC.

During transcription, many steps of mRNA synthesis and processing are integrated through the C-terminal domain (CTD) of the largest subunit of RNAPII (RPB1). A hallmark of RPB1 in most eukaryotes is the presence of a repeating heptapeptide motif in the CTD [14]. In most eukaryotes, the heptad repeat has the consensus sequence YSPTSPS and is present in many copies ranging from 52 in humans to 26 in *Saccharomyces cerevisiae*. Moreover, the number and sequence of repeats is highly invariant within and even between species. By contrast, the CTD of *Plasmodium* spp. differs by the inclusion of a lysine at position 7 of the heptad repeat (YSPTSPK), contains fewer repeats and shows much greater variability in repeat number between and within *Plasmodium* species [15, 16].

Among other modifications, the serine residues at positions 2 and 5 (and 7 in many organisms), can be phosphorylated and intensive effort has gone into trying to understand the role of these modifications in gene expression. Much attention has focused on the functional consequences of an unphosphorylated CTD, mono-phosphorylation at position 5 (Ser5-P), and di-phosphorylation at positions 2 and 5 (Ser2/5-P). A long-held model proposes that the enzymatic activity of RNAPII is determined by the phosphorylation status of the CTD. In this model, RNAPII bearing a hypophosphorylated CTD is enzymatically inert, while Ser5-P is required for RNAPII to initiate transcription, and Ser2/5-P then confers the elongating and highly processive activity of RNAPII [14]. However, a revised model suggests that the phosphorylation status of the CTD may simply be a correlative marker of RNAPII activity [17]. Thus, while the exact functions of phosphorylation events at the CTD are a matter of debate, there is a strong consensus that the presence of Ser5-P and especially Ser2/5-P are marks of transcriptionally active polymerase.

We have exploited the phosphorylation state of the RNAPII CTD to assess the engagement of most *P. falciparum* genes with the transcriptional machinery across the IDC. This allowed us to assess the extent to which RNAPII occupancy correlates with the mRNA accumulation. Our data indicate that *P. falciparum* genes are divided into two classes depending on whether peak RNAPII binding occurs early or late during the IDC. When comparing RNAPII occupancy to mRNA abundance, we identified subsets of genes with high occupancy and relatively low RNA levels and *vice versa*. We find that genes at which RNAPII is detected early (8 to 32 hours post-infection (hpi)) are shorter in length, have more introns and shorter first exons when compared to genes at which RNAPII is detected late (40-48 hpi). Furthermore, we find distinct RNAPII occupancy patterns for the early and late genes along the gene length. Comparison of RNAPII occupancy with various histone modifications identifies chromatin changes that precede, accompany and follow RNAPII recruitment. Together our results show that occupancy by presumed active forms of RNAPII cannot account for the highly orchestrated pattern of mRNA accumulation and loss over the IDC. We suggest that post-initiation mechanisms such as transcriptional elongation and mRNA stability may play predominant roles in determining the dynamics of mRNA abundance during infection of red blood cells.

## Results

### Production of monoclonal antibodies specific for three forms of the CTD of the large subunit of RNAPII

**Figure 1:**
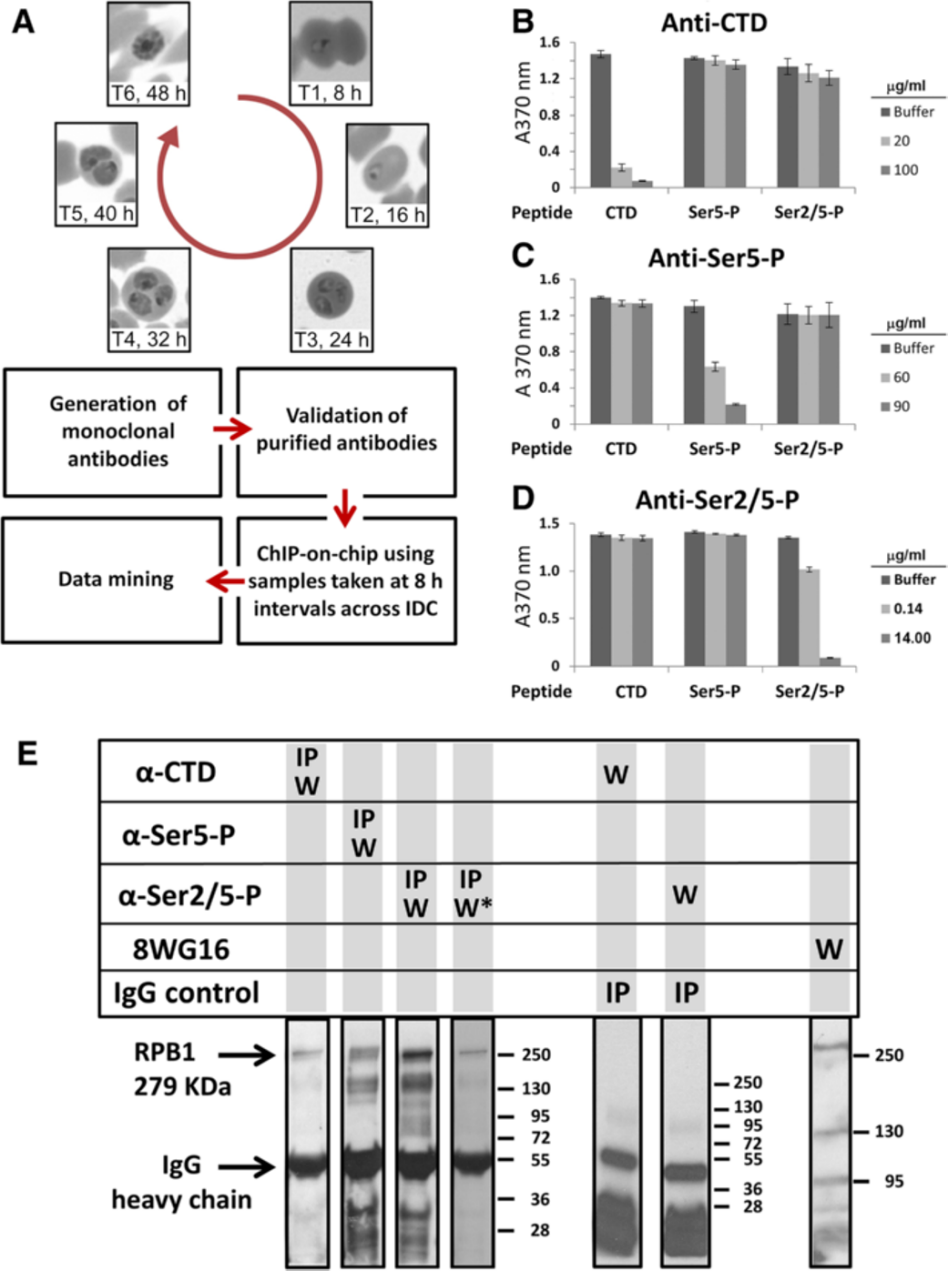
**Validation of monoclonal antibodies against RNA polymerase II. (A)** Schematic of the work flow. *P. falciparum* samples were harvested every 8 h across the 48 h life-cycle at time points (TP) 1 through TP6. The same culture was used for triplicate ChIP-on-chip and transcriptome analyses. **(B–D)** Peptide competition assays. Monoclonal antibodies were raised against peptides corresponding to the unmodified heptad repeat (anti-CTD), the heptad repeat monophosphorylated at position 5 (αSer5-P) and diphosphorylated at positions 2 and 5 (αSer2/5-P). Each monoclonal antibody was pre-incubated with each of the three immunogenic peptides derived from the RNAPII CTD. Reactivity against the immunizing peptide was then tested by ELISA. The concentration of competing peptide in μg/ml is given to the right of each graph. **(E)** Parasite nuclear protein lysate from an asynchronous culture was immunoprecipitated (IP) with each of the three monoclonal antibodies raised in this study or a non-specific IgG control. The precipitates were separated by 6% SDS-PAGE and Western blot (W) performed with the indicated antibodies. The appropriately sized band for *P. falciparum* RPB1 (279 KDa) was observed with all three antibodies (arrowhead). Two panels are shown for antibody α-Ser2/5-P and represent the same Western blot following a moderate and more extensive (W*) series of washes. Note that the only significant specific band is of the correct molecular mass for RPB1. No specific bands are detected by the α-CTD and α-Ser2/5 antibodies following IP with control IgG. The commercial anti-CTD antibody 8WG16 detects a band of the correct molecular mass for *P. falciparum* RPB1 along with several faster running species. The positions of the marker bands in KDa are given to the right of the panels they describe.

Steps in the generation and use of monoclonal antibodies in this study are summarized in Figure 1A. To examine the genome-wide distribution of RNAPII, we raised highly specific monoclonal antibodies against three forms of the *P. falciparum* RPB1 CTD: unmodified CTD, Ser5-P-bearing CTD, and CTD having the Ser2/5-P double phosphorylation. The phosphorylated forms of RPB1 have been associated with RNAPII molecules that are functionally engaged with bound genes. Unphosphorylated heptad repeats may be associated with inert forms of the polymerase, but could also be present as one or more copies within a CTD that also contains phosphorylated motifs. The specificity of the antibodies was confirmed in several ways. First, the antibodies showed high specificity in ELISA by serial dilution (data not shown) and, second, by competition with the immunizing peptide in a peptide competition assay (Figure 1B-D). These results establish that each antibody specifically recognizes its cognate peptide and not the other two. Third, the appropriate sized band for *P. falciparum* RBP1 is also observed on Western blot of nuclear lysates following immunoprecipitation using each of the three monoclonal antibodies (Figure 1E). A well-characterized commercially available antibody (8WG16) that recognizes unphosphorylated and Ser5-P CTD [18] likewise detects a similarly sized band corresponding to RPB1 in a Western blot of cell lysate. Although all antibodies (including 8WG16) detect additional bands, stringent washing leaves a unique band corresponding to the expected molecular weight of RPB1 as the major species detected by the αSer2/5-P antibody (indicated by W* in Figure 1E). Fourth, IP followed by mass spectrometry revealed that both the αSer5-P and αSer2/5-P antibodies precipitated RPB1 as well as two additional subunits of RNAPII, namely RPB2 and RPB3, with high confidence. No RNAPII subunits were precipitated by a control IgG (Additional file 1: Table S1). Together, these results show that these antibodies are specific for the CTD of RPB1 in *P. falciparum* and are suitable for use in the chromatin immunoprecipitation assay (ChIP).

### RNA polymerase II shows a bi-phasic binding distribution across the *P. falciparum*IDC

We next used these *P. falciparum* anti-RNAPII antibodies in ChIP-on-chip analysis to map the position and enzymatic status of RNAPII across the entire *P. falciparum* genome over the 48 h of the IDC at 8 h intervals. The commercial anti-CTD antibody, 8WG16, was also used in parallel. We used custom-made microarrays spotted with 70-mer oligonucleotides covering both coding and intergenic regions [19, 20].

**Figure 2:**
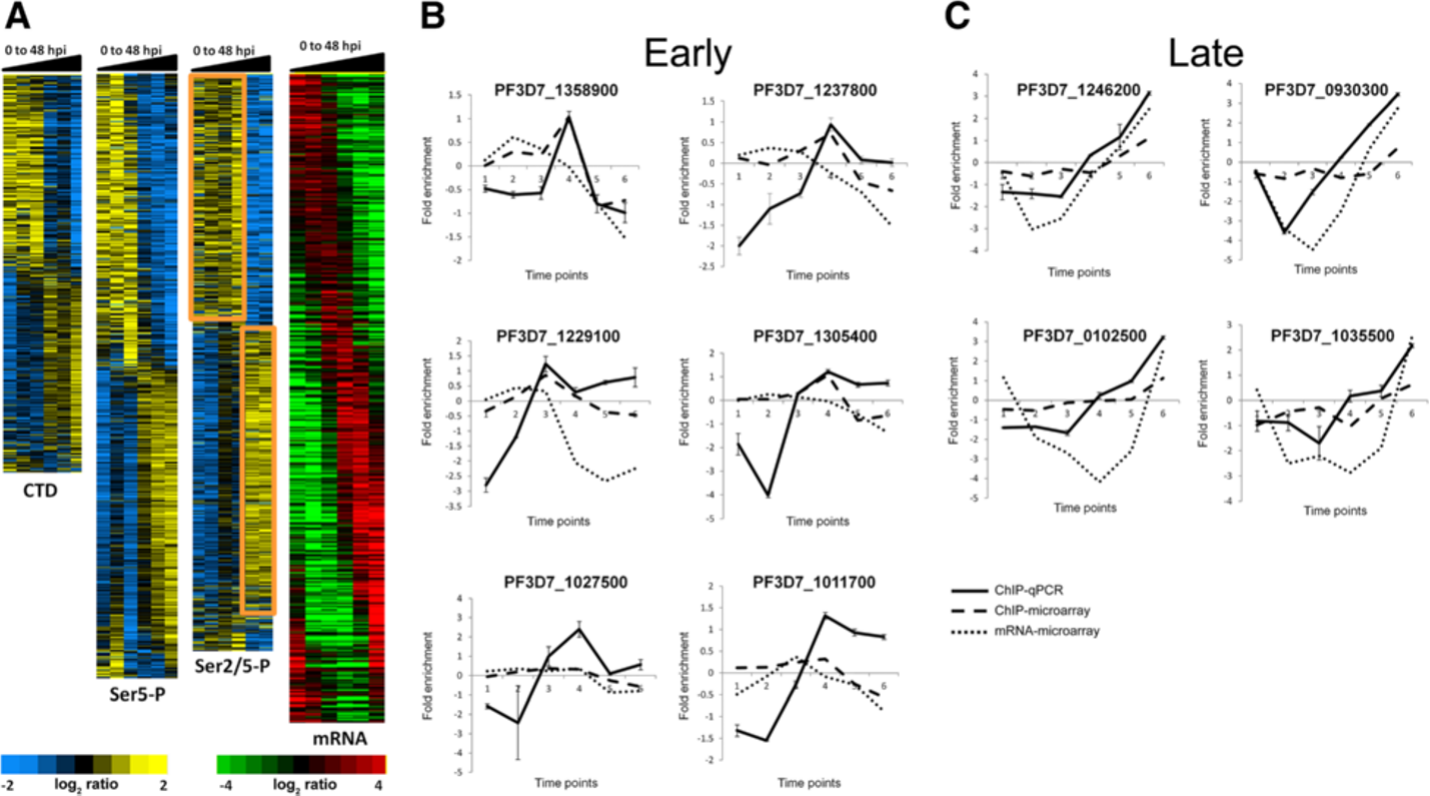
**ChIP-on-chip reveals two classes of genes distinguished by recruitment of RNAPII either early or late in the IDC. (A)** Three phaseograms (blue-yellow scale) representing temporal occupancy patterns for the three forms of RNAPII detected in this study: unphosphorylated CTD, Ser5-P and Ser2/5-P. The data were filtered to include only those loci where RNAPII shows an oscillating profile with *p* <0.05 and a fold change ≥1.5 across the life cycle. The yellow-blue color scale represents the mean-centered log transformed ratios of ChIP/input values across the IDC (T1 to T6). Two prominent probe clusters binding the Ser2/5-P form of RNAPII early vs late in the IDC are marked with orange rectangles. The phaseogram on the far right (green-red scale) depicts the phase of peak gene expression for the *P. falciparum* transcriptome across the IDC. **(B-C)** ChIP-qPCR confirms the presence of two classes of genes distinguished by enrichment in RNAPII binding either early or late in the IDC. Using immunoprecipitated DNA, quantitative real time PCR was carried out on representative genes chosen from the early **(B)** and late **(C)** categories to validate the ChIP occupancy profiles of Ser2/5-P RNAPII across the IDC. Primers for real time PCR were designed to amplify 200–300 bp regions around the respective probe on the microarray. The x-axis gives time points T1 to T6 and the y-axis shows the fold change. Solid lines represent real time PCR profiles, whereas dashed lines give the microarray (ChIP-on-chip) profile at the same probe region. The fine dotted line represents the mRNA levels across the six time points determined by hybridization of cDNA to the microarrays. Error bars show standard deviation of triplicate determinations. See also Additional files 2, 3 and 4: Figures S1, S2 and S3.

Following hybridization and analysis of the microarrays, we selected all those probes exhibiting a significantly oscillating profile with *p* <0.05 and ≥1.5 fold change across the life cycle. This cut-off resulted in the retention of a subset of probes for each of the three antibodies: 2,936 probes with the αCTD (unphosphorylated) antibody; 4,462 probes with the αSer5-P antibody; and 4,255 probes with the αSer2/5-P antibody. We addressed the temporal order within the dataset by creating a phaseogram of RNAPII occupancy*.* The genome-wide profiles reveal a striking periodicity involving the majority of genes (Figure 2A). Most genes can be divided in two groups: those that bind RNAPII early during the IDC and those that bind RNAPII late, regardless of the phosphorylation state of the CTD. This division into early versus late is most dramatic for the Ser2/5-P form of RNAPII which is the elongating form of the polymerase. The data show a striking switch between T4 (32 hpi) and T5 (40 hpi) such that the Ser2/5-P form binds maximally to one large fraction of genes early in the IDC until T4 (1,600 genes), but then switches to a largely mutually exclusive set of genes late in infection during T5 and T6 (1,359). Only 11% of genes are present in both categories due to differential signals from probes within the same gene. A similar observation is made for data sets obtained with the other two antibodies against the unmodified CTD and the Ser5-P; however, the switch between early and late binding profiles for these two forms occurs earlier between T3 (24 hpi) and T4 (32 hpi). Differential occupancy of the different forms of RNAPII argues against a bias inherent to the experimental procedure or the microarrays themselves. Importantly, ChIP-on-chip with the commercial 8WG16 antibody likewise revealed a segregation of early *vs* late signals for the majority of probes in a pattern resembling those obtained with the α-CTD and α-Ser5-P antibodies (Additional file 2: Figure S1A). This is consistent with the demonstration that 8WG16 recognizes the unmodified and Ser5-P forms of the heptad repeat [18]. Hierarchical clustering of the datasets also highlights the distinct distribution of RNAPII in just two prominent groups, one dominated by loci where RNAPII binds early in the IDC and another dominated by loci where the binding is late (Additional file 2: Figure S1A and Additional file 3: Figure S2). The timings of the switches are identical to those revealed in the phaseograms.

To confirm results obtained by microarray, we performed ChiP-qPCR at each of the six time points for six genes that bind the elongating (Ser2/5-P) form of RNAPII early in the IDC and four that bind late. Although qPCR reveals a greater dynamic range than the microarray analysis, the results show that all early genes acquire peak RNAPII recruitment early in the IDC at either time point 3 or 4 (24 and 32 hpi), while late genes do not display maximal RNAPII binding until time point 6 (48 hpi) (Figure 2B,C). These findings strongly validate the results of ChIP-on-chip and confirm the presence of two gene classes distinguished by early or late enrichment in RNAPII binding over the course of the IDC.

### Relationship between transcriptional activity and mRNA abundance

**Figure 3:**
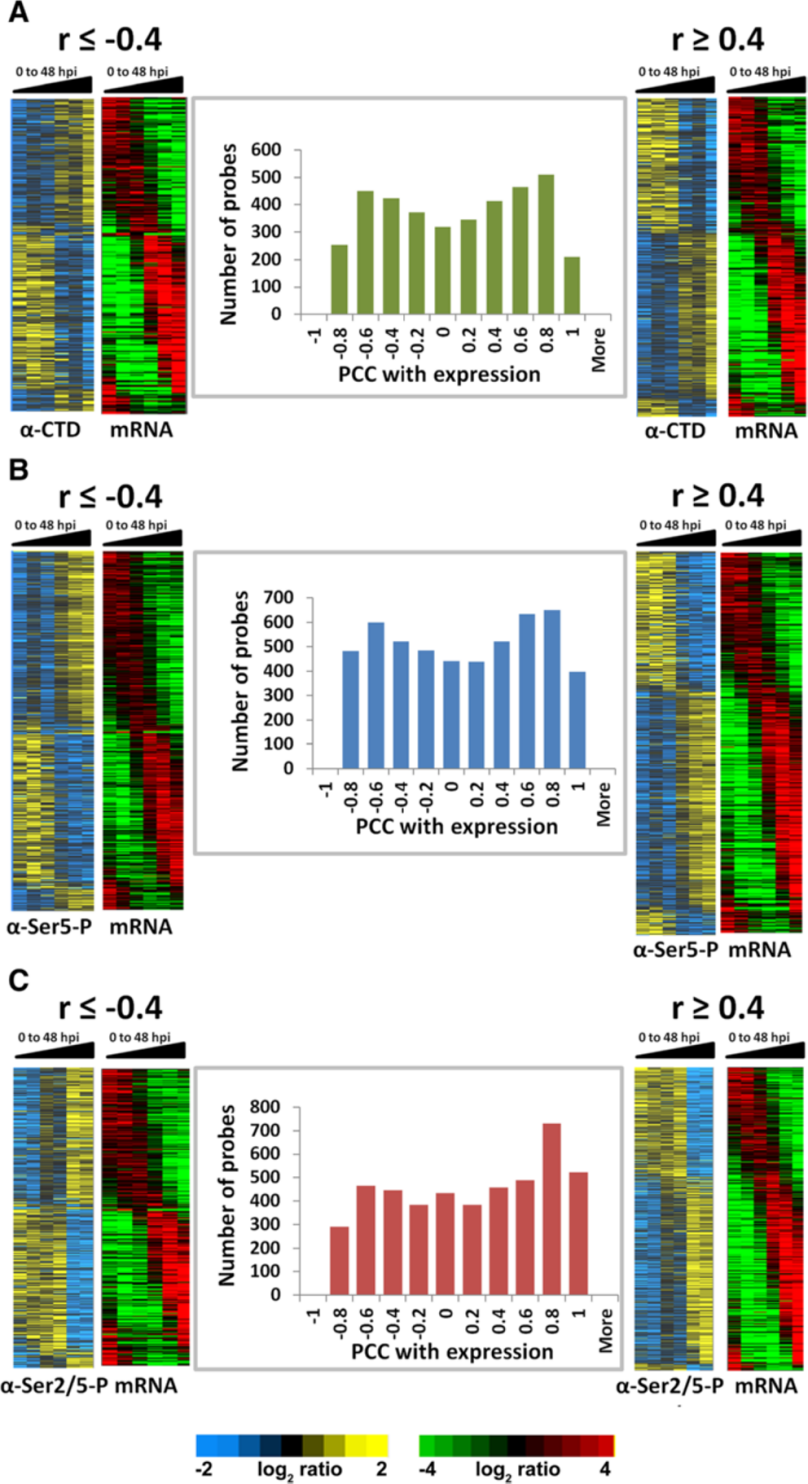
**Relationship between RNAPII occupancy and mRNA abundance.** Correlation between the genome-wide detection of unmodified CTD **(panel A)**, Ser5-P **(panel B)** and Ser2/5-P **(panel C)** forms of RNAPII and mRNA levels was calculated using Pearson’s correlation coefficient (PCC) between the mean-centered profile of all probes with *p* <0.05 and the corresponding gene transcripts. The data have been presented as bar graphs with bins of PCC ranging from −1 to +1 on the x-axis plotted against the number of probes falling into each bin on the y-axis. The phaseograms in the right-hand panels represent probes which are highly positively correlated with gene expression (r ≥0.4), and in the left-hand panels display probes showing negative correlation with gene expression (r ≤ -0.4). One-third of the probes do not show any correlation. The order of the probes represented in the phaseograms for RNAPII binding is the same as those used in the mRNA phaseogram. On the basis of this analysis, we identify four subsets of genes with respect to the correlation between RNAPII binding and mRNA abundance. These are early/positive (RNAPII binds early in the IDC and is positively correlated with mRNA levels), early/negative and late/positive and late/negative.

To characterize the relationship between the genome-wide binding of distinct forms of RNAPII and mRNA abundance, we calculated the Pearson’s Correlation Coefficient (PCC or r) between the dynamic RNAPII occupancy (p <0.05 and 1.5 fold change) and the expression levels of corresponding mRNAs. We established two subsets (Figure 3). In one subset, increased RNAPII occupancy (yellow) was positively correlated with high mRNA levels (red). In the other subset, the correlation was negative, meaning that there was either high mRNA expression without detectable RNAPII binding, or that we observed RNAPII binding without corresponding mRNA expression. With respect to the Ser2/5-P isoform of RNAPII, 1,740 probes were highly positively correlated with gene expression (right panel), while 1,200 probes were strongly negatively correlated (left panel) (Figure 3C). Similar observations were also made with the unmodified CTD and Ser5-P isoforms of RNAPII (Figure 3A,B) as well as with the 8WG16 commercial antibody (Additional file 2: Figure S1B). Approximately a third of the probes showed no significant correlation. The above results thus reveal four subsets of genes with respect to RNAPII occupancy and mRNA abundance: genes which bind RNAPII early and are either positively or negatively correlated with mRNA levels (early/positive and early/negative), and genes which bind RNAPII late (late/positive and late/negative).

We assessed the genomic characteristics of RNAPII-Ser2/5-P-associated early and late genes. We mapped the genes that showed either early or late binding of RNAPII Ser2/5-P onto the 14 *P. falciparum* chromosomes. All chromosomes were seen to bear potential clusters (Additional file 4: Figure S3); however, closer examination revealed that genes within such clusters do not share a common expression profile, arguing against any functional organization and consistent with a previous study suggesting that *P. falciparum* lacks chromosomal domains with common expression profiles [1]. We mapped several genomic descriptors known to associate with gene expression regulation in human [21], including gene length, GC content, number of introns and intron length. We found that genes binding RNAPII Ser2/5-P at early times (T1 to T4) are short with lower GC content by comparison to late genes (T5 and T6) which are significantly longer (Additional file 5: Figure S4A, B). In addition, the early genes had a greater number of introns when compared to late genes (Additional file 5: Figure S4C; see below). The presence of introns has been linked to higher transcriptional efficiency [22]. We assessed the length of the first exon and found that late genes have longer first exons when compared to early genes (Additional file 5: Figure S4D). Thus, early-transcribed genes are shorter in length, have more introns and shorter first exons. Short gene length and short first exons are properties of highly expressed genes in human cells [23, 24].

### Differential distribution of RNAPII along the transcription unit distinguishes genes binding RNAPII early vs late in the IDC

**Figure 4:**
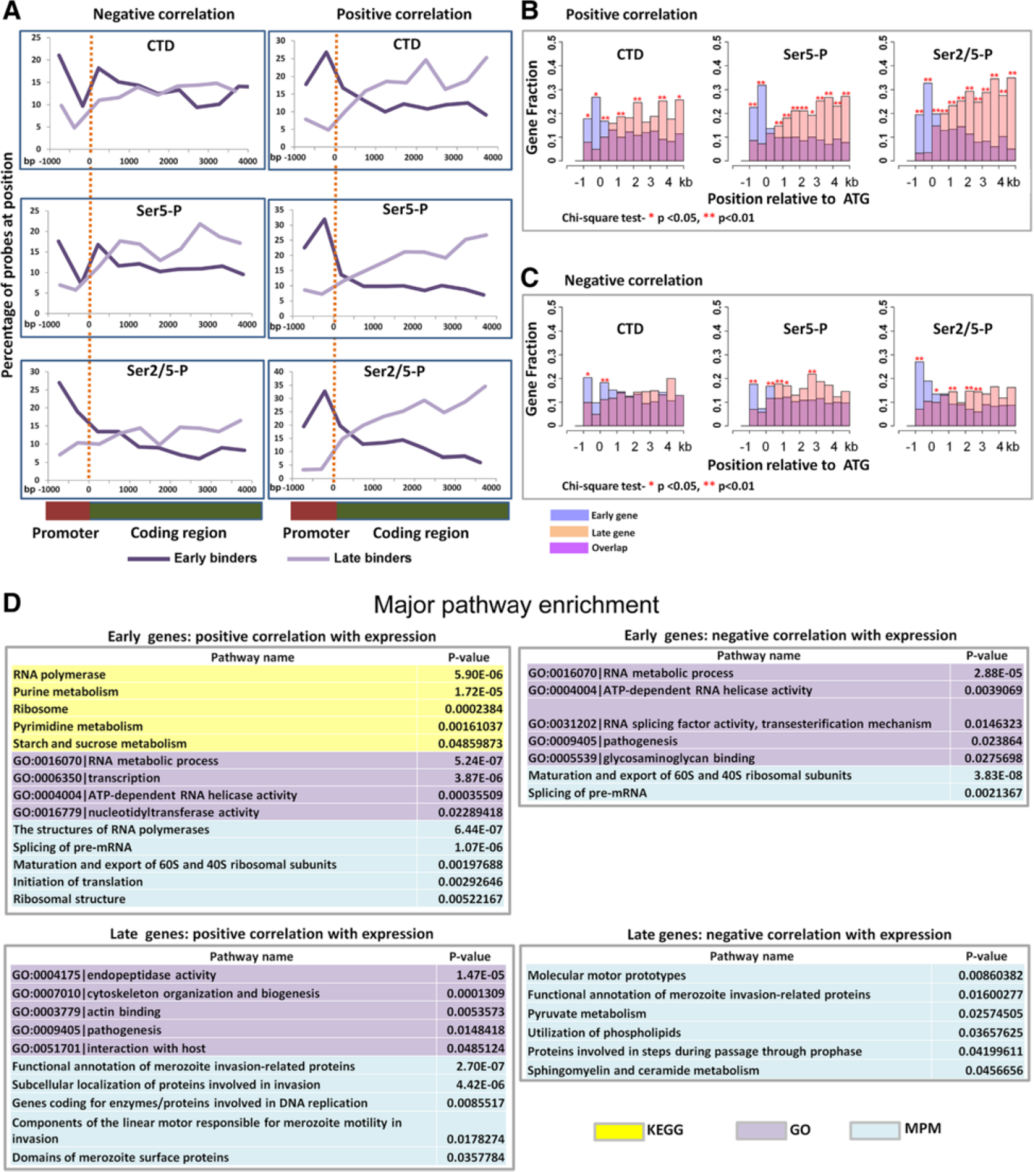
**Distribution of RNAPII along the gene length and enriched pathways. (A)** The plots represent the average distribution along the gene of probes (with *p* <0.05) associated with each form of RNAPII. The start codon is taken as “0”. Data are organized into bins ranging from -500 bp upstream of the ATG to +4000 bp downstream and plotted against the percentage of total probes falling into each bin. The position of a probe along the *x* axis is the average of the positions of all probes within a given bin. While all genes are aligned at the ATG (“0”), they terminate at a wide range of positions downstream with an average at +2.5 kb. The plots on the right show the distribution of early/positive and late/positive loci (r >0.4), while those on the left plot the distribution of early/negative and late/negative loci (r <0.4). **(B-C)** The plots represent the average distribution of the genetic loci associated with each form of RNAPII as a function of gene length. Position distributions were plotted as histograms for each gene group with intervals of 500 bp from -2 kb to +5 kb. Histograms were plotted to display the binding positions in 500 bp intervals from -2 kb to 5 kb flanking the translational start codon at 0. A goodness-of-fit test was performed within each bin to calculate the probability of that region being equally bound by RNAPII for different gene groups. Regions with binding bias by group were assigned *p* <0.05 using the chi-square test. Data were assessed in this manner for **(B)** early/positive and late/positive and **(C)** early/negative and late/negative subsets. **(D)** Functionally enriched pathways (*p* <0.05) for each of the four gene groups. Colors refer to the three distinct databases that were used for pathway enrichment analysis.

According to one model of CTD function, phosphorylation of Ser5 residues predominates near the promoter, whereas toward the end of the gene RNAPII is extensively phosphorylated on Ser2 residues [25, 26]. To explore the phosphorylation status of the CTD at different positions along the transcription unit, we examined the distribution of the three forms of RNAPII along the gene for each of the four classes, namely early and late binders that positively correlated with the mRNA levels (Figure 4A, right panels) and early and late binders that negatively correlated with mRNA levels (Figure 4A, left panels). We observed very distinct profiles for genes that show a positive correlation between mRNA levels and peak RNAPII binding either early or late in the IDC. Promoter regions in *P. falciparum* are predicted to lie between 300 and 1650 bp upstream of the ATG [27]. RNAPII accumulates in the promoter region of early binders, whereas for late binders RNAPII is enriched in the gene body (Figure 4A, right panel). Likewise, the Ser2/5-P form of RNAPII was concentrated within upstream presumptive promoter regions (–1,000 to –500) of genes expressed early and negatively correlating with mRNA levels (Figure 4A, left panel). On the other hand, the unmodified (CTD) and Ser5-P forms did not obviously follow this distribution. A goodness-of-fit test assigns a significant *p* value (<0.01) to the difference in RNAPII distribution (all three forms) between early and late binders (Figure 4B,C) suggesting that the transcriptional process in early vs late phases may be distinguished by mechanistic differences. However, it is possible that this has more to do with the transcription of shorter vs longer genes or the coupling of the splicing machinery to the CTD, since “late” genes are longer and have fewer introns, as noted above.

To determine whether the observed differential recruitment of RNAPII at early vs. late times of infection also reflects functional gene classes, we mapped early and late genes to known pathways and determined which pathways are over-represented in each group by using three pathway databases, namely Gene Ontology (GO), Kyoto Encyclopedia of Genes and Genomes (KEGG) and Malaria Parasite Metabolic Pathway (MPMP) (Figure 4D). We found that early genes were significantly enriched at loci associated with growth (ribosome structure and maturation), metabolism (starch and sucrose metabolism, purine and pyrimidine metabolism), and transcription and splicing. As expected, late genes were significantly enriched in proteins involved in host-parasite interactions and pathogenesis (merozoite invasion and motility, merozoite surface proteins) and cytoskeleton organization and biogenesis.

### Relationship between RNAPII occupancy and chromatin modifications

**Figure 5:**
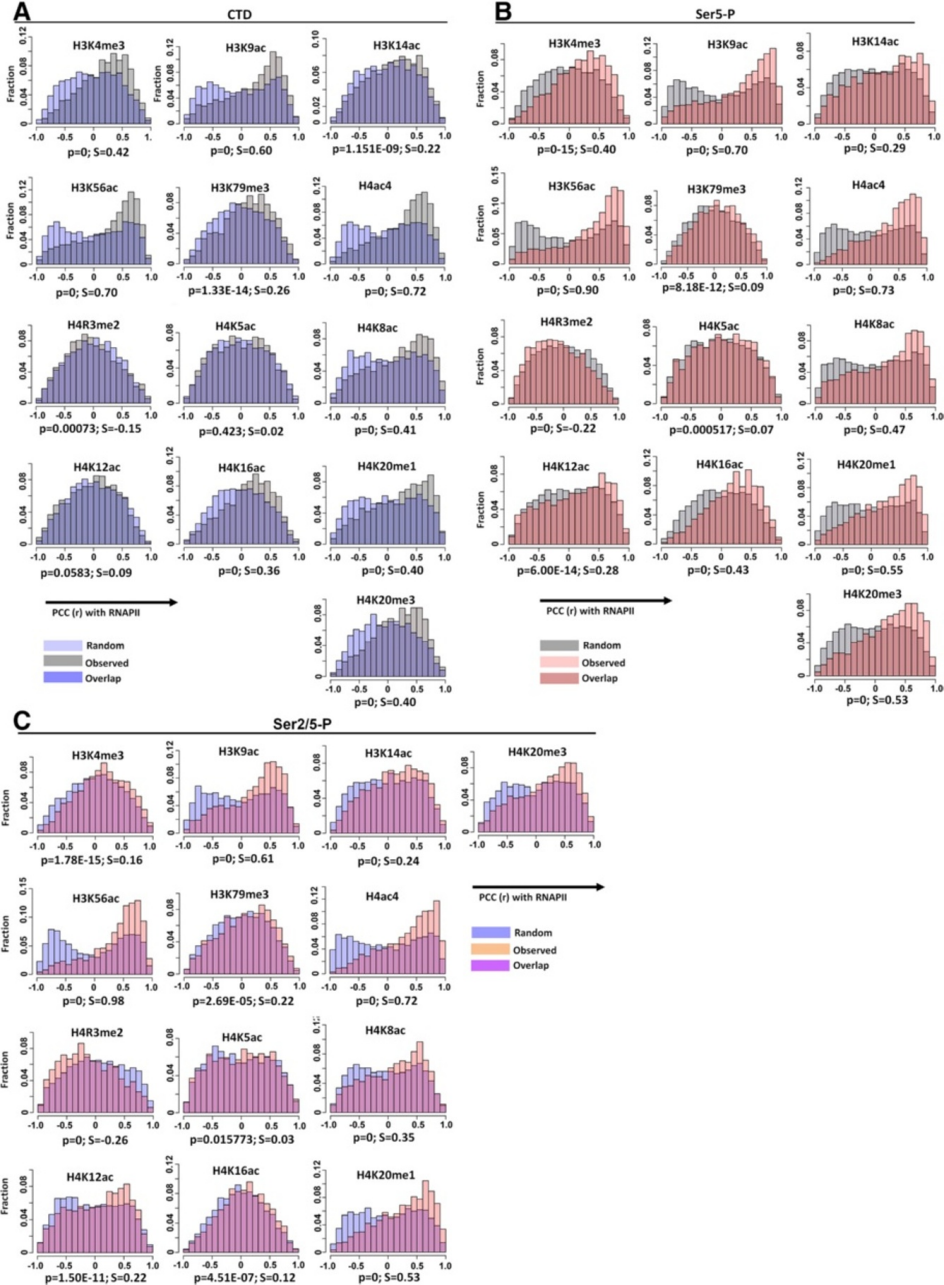
**Relationship between RNAPII occupancy and chromatin modification. (A-C)** Distribution of correlation between the binding of the three forms of RNAPII and 13 different histone mark occupancy profiles for ChIP-enriched probes showing an oscillating profile with *p* <0.05 and ≥1.5 fold change across the IDC. Correlation distributions were plotted as histograms for each histone modification, with bins of PCC ranging from −1 to +1 plotted against the fraction of probes falling into each bin (the number of probes falling into each bin over the total number of probes assessed) for random vs. observed distributions. A Kolmogorov-Smirnov test was applied to compare observed and random correlations, and the statistical significance (*p*) of the observed distribution can be seen at the bottom of each graph. Modified degree of skewness (S) of the PCC distribution is given on the bottom right of each graph (see Methods). See also Additional files 5, 6 and 7: Figures S4, S5 and S6.

Our group and others have shown that chromatin influences gene expression in *P. falciparum* [6, 19, 28–34]. To examine the relationship between RNAPII occupancy and chromatin modification, we calculated the PCC (r) between dynamic RNAPII occupancy of unmodified CTD, Ser5-P and Ser2/5-P and 13 histone modifications determined in our previous study [19]. We identified 5 histone marks including H3K9ac, H3K56ac, H4ac4, H4K12ac and H4K20me1 which showed clear positive correlation with Ser2/5-P RNAPII binding at high statistical confidence (Figure 5). All these histone modifications, in addition to H3K4me3, associate with transcriptionally active protein coding genes and are described as active marks [30, 31, 33], and references therein.

**Figure 6:**
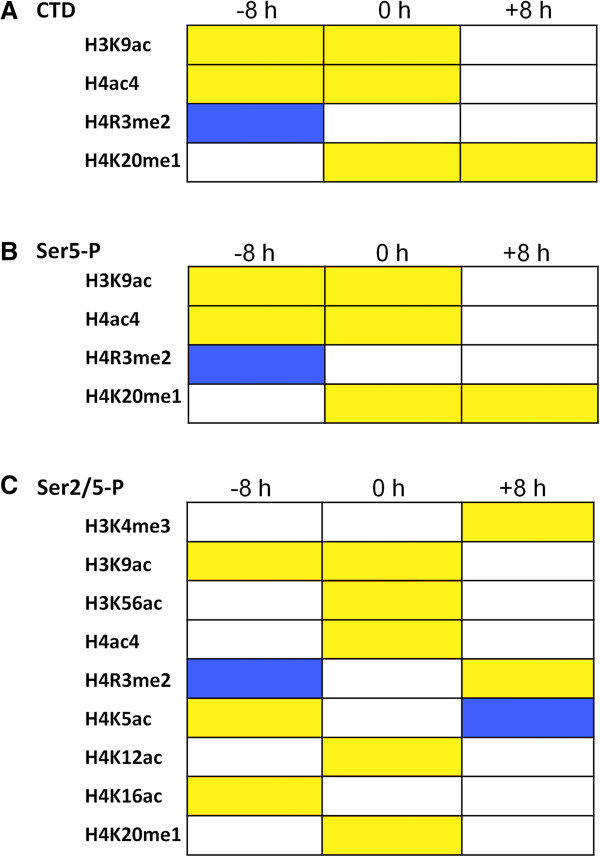
**Statistically significant correlations between RNAPII binding and histone modification**. Binding of the **(A)** unmodified (CTD), **(B)** Ser5-P and **(C)** Ser2/5-P forms of RNAPII was correlated (PCC) with 13 histone modifications present either 8 h earlier (-8 h) or 8 h later (+8 h) than RNAPII binding. A two-sample Kolmogorov-Smirnov test was applied to each pair of data to test whether the function of correlations between RNAPII occupancies and histone modifications were less or greater than the function of similar correlations after 8 h shift in RNAPII or histone modification profile. For each histone modification, a mode of correlation (mc) greater than 0.4 is shown in yellow, whereas an mc less than -0.4 is in blue. A white square denotes no correlation. The correlations derived from comparison within the same time point (0 h; see Figure 5) are included for convenience. Only statistically significant correlations are shown.

To determine whether some histone modifications may precede or follow RNAPII recruitment, we calculated the PCC (r) between RNAPII occupancy (all three forms) and histone modification occurring 8 h before or after recruitment (Figure 6). For example, recruitment of each form of RNAPII at T2 (16 hpi) was assessed relative to histone modifications occurring 8 h earlier at T1 (8 hpi), and 8 h later at T3 (24 hpi), and so on for all six time points. For all forms of RNAPII, we found that some histone marks precede, accompany or follow RNAPII recruitment. Thus, the activating histone mark H3K9ac precedes (and accompanies) recruitment of all forms of RNAPII (Figure 6A-C). Other marks are specific for one or more of the RNAPII isoforms. Thus, H4K16ac, a mark of actively transcribed euchromatin [35], and H4K5ac which has been shown to “bookmark” genes for rapid activation [36], show clear positive correlations with subsequent recruitment of the Ser2/5-P form of RNAPII (Figure 6C). By contrast, both H3K4me3 and H4R3me2 show positive correlation with these histone modifications following recruitment of Ser2/5-P RNAPII. Conversely, H4R3me2, associated with repression of gene expression [37, 38], shows clear negative correlation with subsequent recruitment of all forms of RNAPII (Figure 6A-C), while H4K5ac was depleted 8 h following recruitment of the RNAPIISer2/5-P form (Figure 6C). Both the positive and negative correlations are highly statistically significant by the Kolmogorov-Smirnov test (p <0.001). Other histone modifications were not seen to anticipate or follow RNAPII recruitment (Additional files 6, 7 and 8: Figure S5, S6 and S7).

## Discussion

In this study, we performed genome-wide mapping of RNAPII occupancy over the 48 h of the IDC using a ChIP-on-chip approach. We used three highly specific monoclonal antibodies against different forms of the *P. falciparum* RBP1 CTD in addition to 8WG16, a well-characterized commercial monoclonal antibody raised against wheat germ RNAPII and likewise recognizing the CTD [18, 39]. All four antibodies gave highly similar results. We observed that the parasite’s IDC is divided into early and late phases of RNAPII occupancy. Maximal binding by the forms of RNAPII bearing unmodified and Ser5-P modified heptads (recognized by α-CTD, α-Ser5-P and 8WG16) switches from one large set of genes to another between T3 (24 hpi) and T4 (32 hpi), while that of the Ser2/5-P form does so between T4 and T5 (40 hpi). This is the first indication of which we are aware that the *Plasmodium* life cycle is divided into rather stark early vs. late stages defined by a component of the transcriptional machinery.

Considerable controversy has surrounded the role of the CTD heptad repeats and their phospho-serines. While the heptad repeats are necessary for the survival of yeast and human cells, they are dispensable for transcription *in vitro* [40] *.* In addition, the loss of Ser2 phosphorylation does not affect the transcription of the majority of genes in fission yeast, while the budding yeast kinase Kin28p is not required for transcription but rather to couple transcription initiation to the capping process [40]. These and other observations have led to the suggestion that the heptad repeats are not required for transcription *per se*, but rather to bind and coordinate the activity of proteins exerting co-transcriptional functions such as capping and splicing [17]. Apicomplexans are within the branch of eukaryotes stemming from an ancestor bearing CTD heptad repeats. However, the severe reduction in repeat number in basal *Plasmodium* species suggests that heptad repeats were first lost in *Plasmodium* spp and then re-acquired in primate-infecting lineages [15, 16]. This raises the possibility that the heptad repeats of *P. falciparum* play roles entirely unrelated to those of their counterparts in other species, or at least unrelated to transcriptional processes. This seems unlikely for several reasons. First, *P. falciparum* encodes an almost complete set (13 of 14) of so-called core CTD-interacting proteins with major transcriptional functions [41]. Second, *P. falciparum* encodes a clear homolog of the yeast Ctk1 kinase in part responsible for Ser2 phosphorylation (PF3D7_0417800) [41, 42] and another kinase displaying striking similarity to human CDK7 responsible for phosphorylation at Ser5 (PF3D7_1014400; E =3e-76 by BLASTP). Third, as revealed by our antibodies, both serines 2 and 5 of the *P. falciparum* CTD are phosphorylated *in vivo*. Fourth, an alternative register and alignment of the functional heptad unit suggests that basal *Plasmodium* species may harbor more units of full and partial heptads than previously appreciated [43], suggesting that interactions between the CTD and its partners were never lost in these organisms. Fifth, the evolution of exclusively new functions for the CTD should be accompanied by the evolution of a new motif, not the same heptad motif already implicated in earlier functions. Together, these observations argue that the evolution of a completely new set of functions for the heptad repeats is less parsimonious than the re-establishment (or enhancement) of interactions with partners previously selected in the course of evolution. The above argument does not preclude, however, that the *P. falciparum* CTD may have also acquired novel functions and interaction partners.

At a minimum for the interpretation of our results, it seems reasonable to expect that the presence of phosphorylated serines in the CTD is indicative of RNAPII that is actively engaged in transcription. We find a similar distribution of RNAPII bearing unmodified and phosphorylated heptads. Similar observations have been made by several groups and can be explained simply by the presence of one or more unmodified heptads in the company of other phosphorylated repeats within a CTD [17]. Because all the antibodies used in this study find their epitopes in the CTD, it remains possible that we have failed to detect RNAPII sub-populations bearing alternative modifications of this domain. The use of an anti-RNAPII antibody directed against a domain that is not subject to extensive modification and which is evolutionarily stable would therefore provide a useful complement to our results.

It may be particularly insightful to compare our results with those of two previous studies. One report used the nuclear run-on assay to assess the engagement of RNAPII at several genes across the IDC [10]. Strikingly, some genes showed an inverse correlation between RNAPII engagement and mRNA levels, consistent with our early-negative and late-negative categories in which RNAPII binding, or lack thereof, is not reflected in mRNA levels. In another study, ChIP was used to assess the recruitment of the general transcription factors TBP and TFIIE to a number of genes at both ring (12 – 14 hpi) and trophozoite (32 - 34 hpi) stages (roughly equivalent to our T2 and T4, respectively). Regardless of whether these genes were ring-specific or trophozoite-specific, both TBP and TFIIE were present at these genes at both ring and trophozoite stages [9]. These findings are consistent with our observed discordance between occupancy by RNAPII and the mRNA levels for a large subset of genes. However, whereas we find that the parasite genome is largely divided between those genes that recruit RNAPII early vs late, TPB and TFIIE were found in both rings and trophozoites at genes that are expressed at only one of these stages. At first, these results may seem contradictory, since TFIIE binding is expected to *follow* RNAPII recruitment [44]. However, as described in yeast, a promoter that has undergone one round of transcriptional initiation remains bound to a complex of general transcription factors referred to as the scaffold or re-initiation complex. The scaffold includes TBP and TFIIE, but not RNAPII, and has been proposed to prime the promoter for re-initiation [45]. It is therefore possible that many erythrocyte-expressed genes of *P. falciparum* are primed for re-initiation by a TBP- and TFIIE-containing scaffold, ready for RNAPII recruitment in response to appropriate cues. Two of the genes analyzed for TBP and TFIIE binding are included in our data set. *msp1* (PFI1475w/ PF3D7_0930300) and *rhoph3* (PFI0265c/ PF3D7_0905400) bind TBP and TFIIE at both ring and trophozoite stages [9]. By contrast, we observe RNAPII occupancy at these two genes only late in the IDC: the presence of the unmodified CTD form at *msp1* and the presence of the Ser5-P form at *rhop3* is not observed until T4 (trophozoite stage). Both genes then associate with the Ser2/5-P form by T5. Thus, the appearance of enzymatically active forms of RNAPII *follows* that of TBP and TFIIE, at least in these instances. This is consistent with the presence of TBP and TFIIE in a scaffold-like complex ready for efficient RNAPII recruitment when the time is right.

Our data show that RNAPII occupancy and the corresponding mRNA levels of many genes are well correlated, suggesting that the control of transcription is often a determinant of gene expression. Surprisingly, however, a large number of genes achieve significant mRNA levels only several hours following their peak RNAPII Ser2/5-P occupancy (early/negative and late/negative), suggesting that transcription is not a rate-limiting step at these loci. One possibility is that RNAPII is “pre-loaded” onto such genes but kept in a paused (or poised) state. Although RNAPII levels subsequently fall as mRNA levels rise, this may be due to the greater transcriptional activity of fewer polymerase molecules. This would then place a major regulatory control in *P. falciparum* at the point of transcriptional elongation as has been well documented in other eukaryotes [46]. If so, the parasite must have evolved mechanisms to stall RNAPII despite the presence of the Ser2/5-P modification at the CTD since this form of the polymerase, just as for the unphosphorylated and Ser5-P forms, is enriched at a subset of genes at times when their mRNA levels are low. The dispensability of Ser2 phosphorylation for the expression of most genes in fission yeast is consistent with this notion [47]. Non-exclusively, abundant mRNA despite decreased levels of RNAPII binding could be due to a switch from labile to stable transcript states, consistent with previous studies demonstrating a large increase in transcript stability over the course of the IDC [12].

The highly similar distribution along the gene length of all forms of the polymerase studied here may be consistent with this notion. On the other hand, the apparent distribution of CTD isoforms along the gene in other organisms varies greatly depending on the antibody in use and the presence/absence of modifications adjacent to the epitope (summarized and extended in [17]). From this perspective, the distributions observed in this study may not be surprising. The Ser2/5-P form of RNAPII – sometimes described as the elongating form – is enriched upstream of the coding region of “early” genes. While this may be surprising, a similar distribution for RNAPII phosphorylated at Ser2 of the heptad repeat has been observed for a class of genes in *Schizosaccharomyces pombe* [47].

The division of the IDC into early and late phases as evidenced by RNAPII occupancy profiles is paralleled by the binary changes (open/closed) in chromatin structure that occur during the parasite’s life cycle [48] whereby the chromatin opens at the early ring stage, reaching a maximum at the trophozoite stage followed by tight packing in schizonts. The late recruitment of RNAPII addresses the apparent contradiction that the parasite chromatin is closed in the late schizont stage despite the appearance of late-stage mRNAs. Although some mRNAs detected in the schizont stage could be regulated at the level of stability, our results show that RNAPII recruitment does take place toward the end of the IDC, though restricted mostly to loci involved in late-stage-specific processes such as pathogenesis, merozoite maturation and invasion. Elucidation of the molecular determinants of this early-late transcriptional switch should provide important insights into fundamental aspects of transcriptional control in *P. falciparum*.

We previously carried out genome-wide mapping of 13 histone modifications and correlated their presence at particular genes with transcript accumulation over the IDC. In particular, H4K8ac was tightly linked to mRNA levels and accompanied a promoter inserted at an ectopic site in the genome [19]. Here, we further correlated these histone modifications to RNAPII recruitment and to time points both 8 h earlier and later. The results reveal distinct chromatin signatures preceding, following and at the time of binding of distinct forms of RNAPII. Such signatures may reflect the action of the machinery used in RNAPII recruitment and maturation, but do not obviously provide insights into the mechanism controlling the early/late switch. Interestingly, we did not find a correlation between the peaks of H4K8ac modification and RNAPII binding. And while we do observe a correlation between the binding of the Ser2/5-P form of RNAPII and H3K4me3, maximal levels of this histone mark only follow maximal binding by RNAPII Ser2/5-P. Others have also noted that H3K4me3 is not correlated with transcript levels [6]. These differences in the timings of H4K8ac and H3K4me3 modification, peak mRNA levels and maximal recruitment of RNAPII Ser2/5-P are not consistent with a simple coordinated process of RNAPII binding and transcription immediately facilitated by chromatin modification and leading to increased mRNA output, and further substantiate a role for post-initiation and post-transcriptional processes in the control of gene expression in *P. falciparum*.

## Conclusions

The striking contrast between the relatively simple pattern of RNAPII recruitment (early vs late) to approximately two thirds of genes *versus* the highly orchestrated rise and fall of their transcripts across the IDC suggests that mechanisms downstream of transcriptional initiation may play a predominant role in the control of gene expression in *P. falciparum*. The recent demonstration that the negative regulation of a *var* gene subgroup in *P. falciparum* is mediated by a transcriptionally coupled RNase is consistent with this notion and may have broader implications for gene expression in this species more generally [49]. Limited control at the level of transcription initiation, though rare, would not be unique to malarial parasites. A closely related family of protozoan parasites, trypanosomatids, shows no evidence of developmental control of RNA polymerase II transcription [50]. A bias toward post-initiation regulation of gene expression may have been facilitated by the homeostatically controlled environments within human and insect hosts, thereby relieving selective pressure for the retention of fine control at the level of transcription initiation.

## Methods

### Ethics approval and informed consent

This research involved the use of human blood drawn from healthy volunteers by trained healthcare professionals and was approved by the NTU Institutional Review Board and assigned the IRB approval number IRB-2013-07-020. Informed consent was obtained and documented by the signing of an approved informed consent form.

### Generation of monoclonal antibodies

Peptides based on the heptad repeat of the *P falciparum* RNAPII CTD and bearing (1) no phosphate (CTD)- Biot-Ahx-YSPTSPKYSPTSPK and CYSPTSPKYSPTSPK (2) a phosphate at Ser5 (Ser5-P)-Biot-Ahx-YSPT**S**PKYSPT**S**PK and CYSPT**S**PKYSPT**S**PK or (3) phosphates on Ser2 and Ser5 (Ser2/5-P)- Biot-Ahx-Y**S**PT**S**PK and CY**S**PT**S**PK were used to immunize BALB/c mice. Serine residues in bold typeface were phosphorylated. All three peptides were coupled with Keyhole Limpet Haemocyanin (KLH) using a Imject® Maleimide Activated mcKLH kit. The immunization schedule included a first subcutaneous injection of KLH-coupled peptide resuspended in complete Freund’s adjuvant (CFA, Sigma, St. Louis, USA) followed by a second subcutaneous injection in incomplete Freund’s adjuvant (IFA, Sigma) 6 weeks later. Eight weeks after the first immunization, mice were sacrificed and draining lymph nodes collected. Isolated lymph node cells were fused with SP2/0 myeloma cells using polyethylene glycol (Sigma, St. Louis, USA) according to standard fusion protocols [51]. Cells were resuspended in HAT medium (Sigma), distributed in 96-well plates and wells containing single hybridoma clones were screened by ELISA. All hybridomas testing positive were further subcloned by limiting dilution and retested by ELISA for positivity. N-terminally biotin-tagged peptides were used for application to streptavidin coated plates for ELISA (Pierce). Positive hybridoma clones were expanded in roller bottles and the corresponding monoclonal antibody was purified from the culture supernatant *via* a GammaBind Plus Sepharose (GE HealthCare Life Sciences).

### Peptide competition assay

Phosphorylated and non-phoshorylated peptides were incubated in a range of concentrations with monoclonal antibodies α-CTD, α-Ser5-P and α-Ser2/5-P at room temperature for 30 min. The peptide antibody mix was then tested for reactivity in an ELISA [52].

### Immunoprecipitation and western blots

Parasite nuclear protein extract was prepared by high salt. The nuclear extract was further diluted, pre-cleared and incubated with RNAPII antibodies overnight at 4°C. Protein complexes were precipitated by incubating with Protein G agarose slurry for 1 h at 4°C followed by extensive washings to reduce the background. Protein complexes were eluted by addition of SDS-PAGE loading buffer and boiling for 5 min at 95°C. Supernatants were resolved in 6% SDS-PAGE and subsequently used for Western blot analysis. Western blots were carried out using antibodies raised against the CTD of RNAPII described in this study and the anti-CTD antibody 8WG16 (Covance) [39]. Secondary antibody (anti-mouse-HRP, Sigma) was visualized using a chemiluminescence detection system (Millipore) as per the manufacturer’s instructions.

### Mass spectrometry

Briefly, after immunoprecipitation, the eluent was separated on a SDS-PAGE gel, and silver stained. The gel lane was cut into 5 slices and then processed by a standard in-gel digestion protocol using trypsin. Extracted tryptic peptides of each slice was injected into a LC-MS/MS system (LTQFT Ultra, Thermo Scientific, Bremen, Germany) for peptide sequencing using tandem MS. The MS/MS spectra of the raw data was extracted and converted into dta files using extract_msn and then combined into a mascot generic file using an in-house program as previously described [53].

The *P. falciparum* protein database (5491 sequences, 4147970 residues) was used for database searches [54]. The database search was performed using an in-house Mascot server (version 2.4.1, Matrix Science, Boston, MA, USA) with MS tolerance of 10 ppm and MS/MS tolerance of 0.8 Da. Two missed cleavage sites of trypsin were allowed. Carbamidomethylation (C) was set as a fixed modification, and oxidation (M), and deamidation (N and Q) were set as variable modifications. The obtained peptide/protein list for each fraction was exported to Microsoft Excel or processed using an in-house script for further analysis. Proteins PF3D7_0318200 (PFC0805w; RPB1), PF3D7_0215700 (PFB0715w; RPB2) and PF3D7_0923000 (PFI1130c; RPB3) were positively identified in αSer5-P and αSer2/5-P pull down samples but not with an IgG control antibody. The Mascot results in Excel are provided in Additional file 1: Table S1.

### Parasite culture

*Plasmodium falciparum* T996 strain parasites were cultured as described previously [55]. Highly synchronous parasites were grown at hematocrit of 2% and 5% parasitemia. For ChIP, saponin-lysed parasites were cross-linked with 0.5% formaldehyde and harvested at 8, 16, 24, 32, 40 and 48 hpi. Samples were also taken for RNA isolation from the same time points.

### Chromatin immunoprecipitation

The chromatin immunoprecipitation assay was performed as described [19]. Briefly, parasites were lysed by 200 strokes using a dounce homogenizer and centrifuged. The nuclear pellet was lysed using 1% SDS and the resulting nuclear extract was sheared in Vibra Cell sonifier at 25% amplitude (10 sec on, 50 sec off) for 8 pulses to obtain DNA fragments in the range of 200-1000 bp. The chromatin DNA was diluted, pre-cleared and incubated with the immunoprecipitating antibody overnight at 4°C. RNAPII-antibody-bound complexes were then bound to salmon sperm DNA/Protein A agarose slurry for 1 h at 4°C, followed by extensive washings to reduce the background. Precipitated complexes were eluted from the beads and the protein-DNA cross-links were reversed by heating samples at 65°C overnight in 0.2 M NaCl. DNA was purified using the QIAEX II kit (QIAGEN). Immunoprecipitated DNA and the sonicated genomic DNA (input) were amplified as described [56] with some modifications [57]. Amplified ChIP and input DNA were labeled with Cy5 and Cy3 respectively.

### RNA preparation

For transcriptional profiling by microarray total RNA from all the six time points was isolated using the Trizol method and cDNA was synthesized by reverse transcription, purified and labeled as previously described [1]. Samples for individual time points (labeled with Cy5, red) were hybridized against a reference pool (labeled with Cy3, green) which consisted of a mixture of RNA from all the six time points in equal amounts.

### Microarray hybridization and data analysis

Equal amounts of Cy5 and Cy3 labeled probes were used to probe the microarrays representing the *P. falciparum* genome. These microarrays consisted of14,981 probes, containing 3895 50-mer intergenic oligonucleotide probes and 10,566 70-mer ORF probes covering most of the 5,283 protein-coding genes [20]. On average, probes were spaced every 1,502 bp along the length of most genes as annotated in the *P. falciparum* genomic database (PlasmoDB) [19]. The microarray hybridization was carried at 63.5°C or 65°C in an automated hybridization station (Maui, USA) for ChIP DNA or cDNA respectively as described previously [1]. The microarrays were scanned using the GenePix scanner 400b and GenePix pro 6.0 software (Axon Laboratory). The microarray data were normalized using the Limma package of R [58]. Briefly, lowess normalization was applied to all spots on each array after Edwards background correction (Edwards [58]) and followed by quantile normalization [59] between arrays. Spots with negative flags or with signal intensities less than 1.4 times the background for both Cy5 and Cy3 fluorescence were eliminated. Then, log2 ratio of Cy5 and Cy3 were used to present the RNAPII occupancies for each spot. After averaging triplicates, only spots where the signal from ChIP DNA was obtained in at least 2 experimental repeats and in at least 2 consecutive time points were included in further study. Next, missing values were estimated by Kth nearest neighbor (KNN) imputation using the impute package of R. The estimated values were less than 0.2% of the total. For expression microarray, each gene profile was represented by an expression value calculated as an average of all probes representing a particular gene. Phaseograms for expression and ChIP data were generated by fast Fourier transform method and probes/genes were sorted according to phase from -π to π with the mean-centered log2 ratios. To address the dynamic changes in RNAPII occupancies during the IDC, values for the fold change of RNAPII and a Student’s t-test *p* were assigned to each spot based on comparing the triplicates of summit and valley time points. Significantly oscillated profiles between the summit and valley time points were defined as *p <*0.05 and fold change ≥1.5 across the IDC. For ChIP-chip experiments using the monoclonal antibody 8WG16 exceptionally, microarrays were performed twice and only signals showing ≥1.5 change across the IDC in both arrays were included for analysis.

Pearson’s Correlation Coefficient (PCC or r) was calculated between the RNAPII binding profile and their corresponding mRNA profiles. To compare the distribution of RNAPII along the gene body on the chromosome, histograms were plotted to display the binding positions with 500 bp intervals from -2 kb to 5 kb around coding start site (ATG). A goodness-of-fit test was performed within each bin to calculate the probability of that region being equally bound by RNAPII for different gene groups. Regions with binding bias by group were assigned at *p* <0.05 using a chi-square test.

PCC (or “r”) was calculated between the RNAPII binding profiles and corresponding histone mark profiles. Modified skewness value (S) was also calculated to measure the asymmetry of correlation distribution. Strictly, a negative S value indicates that the distribution is biased toward a positive correlation. However, for convenience, we have taken the negative of the S value such that a positive S value now represents a distribution biased to a positive correlation. Likewise, a negative S value now represents a bias toward negative correlation.

To test how statistically significant the RNAPII levels correlated with a particular histone modification level across IDC, we generated a random dataset which contained ~20 k randomly paired profiles of RNAPII and histone modifications and a two-sample Kolmogorov-Smirnov test was applied to compare the observed correlations and the randomized correlations. In addition, a two-sample Kolmogorov-Smirnov test was also applied to each pair of data to test whether the function of correlations between RNAPII occupancies and histone modifications was less or greater than the function of similar correlations after one time point shift forward or backward in RNAPII profile. In addition, we used the degree of skewness (S) of the PCC distributions as a means of identifying histone marks with maximum probe density showing positive correlation with RNAPII occupancy. We identified 5 histone marks including H3K9ac, H3K56ac, H4ac4, H4K12ac and H4K20me1 with *p* <0.0005 and S >0.05 and which showed clear positive correlation with Ser2/5-P RNAPII binding.

### Quantitative real time PCR

qRT-PCR experiments were performed on immunoprecipiated and input DNA using the SYBR Green PCR master mix (Kappa SYBR Fast Master Mix) according to manufacturer’s instructions. All reactions were run in triplicate. Product-specific amplification was confirmed by performing melting curves for each reaction. For each gene the fold change across the time points was calculated by calculating the difference in the Ct value for each time point.

### Primers

All the primers used in the current study are listed (Additional file 9: Table S2).

### Data access

The microarray data files associated with this study have been submitted to NCBI GEO with accession number GSE57748.

### Additional data files

The following additional data are available with the online version of this paper. Additional file 2: Figure S1) presents the phaseogram, hierarchical clustering and correlations to mRNA levels for ChIP-chip data using the commercial anti-CTD antibody 8WG16. Additional file 3: Figure S2) is a hierarchical clustering of the microarray data presented in Figure 2A. Additional file 4: Figure S3) is a representative example of the chromosomal distribution of genes identified in this study that bind RNAPII either early or late in the infectious cycle. Additional file 5: Figure S4) is an analysis of the genomic features (gene length, GC content, number of introns, first exon length) in the four classes of genes identified in this study. Additional file 6: Figure S5) shows the distribution of correlation between the binding of the unmodified-CTD form of RNAPII and the occupancy profiles of 13 histone modifications assessed before during and after RNAPII binding. Additional file 7: Figure S6) shows the distribution of correlation between the binding of the Ser5-P form of RNAPII and the occupancy profiles of 13 histone modifications assessed before during and after RNAPII binding. Additional file 8: Figure S7) shows the distribution of correlation between the binding of the Ser2/5-P form of RNAPII and the occupancy profiles of 13 histone modifications assessed before during and after RNAPII binding. Additional file 1: Table S1) presents the Mascot results in Excel for results obtained by mass spectrometry with αSer5-P and αSer-2/5 antibodies. Additional file 9: Table S2) is a list of the oligonucleotides used in this study.

## Electronic supplementary material

Additional file 1: Table S1: Mascot results in Excel format following mass spectrometric analysis of peptides co-immunoprecipitated with αSer5-P and αSer2/5-P antibodies. The MS/MS spectra of the raw data was extracted and converted into data files using extract_msn and then combined into a mascot generic file using an in-house program. (XLSX 16 KB)

Additional file 2: Figure S1: Results of ChIP-chip analysis using the commercial anti-CTD monoclonal antibody 8WG16. ChIP-chip was performed in duplicate and only probes with signals showing a fold change ≥1.5 across the life cycle in both experiments were included for analysis. **(A)** Heat maps showing phaseogram and hierarchical clustering of loci bound by RNAPII. **(B)** A comparable analysis to those presented in Figure 3. Approximately one third of probes bound by RNAPII are strongly positively correlated with mRNA levels (*r* ≥0.4) while a distinct third is strongly negatively correlated (*r* ≤ -0.4). (TIFF 818 KB)

Additional file 3: Figure S2: A hierarchical clustering of the microarray data presented in Figure 2A. A hierarchical clustering of log-transformed ChIP/input ratio of RNAPII occupancy for those loci where RNAPII shows an oscillating profile with p <0.05 and fold change ≥1.5 across the life cycle. (TIFF 892 KB)

Additional file 4: Figure S3: A representative example of the chromosomal distribution of genes identified in this study that bind RNAPII either early or late in the infectious cycle. Chromosomal projection of RNAPII Ser2/5-P occupancy for early and late gene groups onto chromosomes 3 (MAL3) and 4 (MAL4). The vertical bars (green) indicate probe position for each gene group. Gene clusters can be seen in red, the results for a “late” cluster on chromosome 4 is shown. Vertical bars denote the positions of late genes, and the bars in red indicate a cluster of 6 such genes spanning a mere 30 kb (shown to scale and with gene names and their transcriptional orientation in the bottom half of the figure). (TIFF 242 KB)

Additional file 5: Figure S4: An analysis of the genomic features (gene length, GC content, number of introns, first exon length) in the four classes of genes identified in this study. Distinct genomic properties of early vs late genes. Box plots show the distribution of **(A)** gene length, **(B)** percentage GC content, **(C)** intron length (intron/exon ratio) and **(D)** length of the first exon for the early and late genes. 1, early/negative, 2, late/negative, 3, early/positive and 4, late/positive. p value for the two group comparisons is calculated using Wilcoxon two-tailed test and the p-value for the four group comparison is calculated using “Kruskal-Wallis test”. (TIFF 1 MB)

Additional file 6: Figure S5: Distribution of correlation between the binding of the unmodified-CTD form of RNAPII and the occupancy profiles of 13 histone modifications assessed before during and after RNAPII binding. Distribution of correlation between the binding of the unmodified-CTD form of RNAPII and the occupancy profiles of 13 histone modifications assessed at 8 h earlier (-8 h) or later (+8 h) than RNAPII binding. PCC was calculated between RNAPII profiles for all the probes with p <0.05 and ≥1.5 fold change and the corresponding histone mark after one-time point shift. For example, RNAPII binding at T2 was correlated to histone marks in place at T1 (-8 h) and T3 (+8 h). **(A)** Correlations with histone H3 modifications. **(B)** Correlations with histone H4 modifications. Correlation distributions were plotted as histograms for each histone modification, with bins of PCC ranging from -1 to +1 on x-axis plotted against the total number of probes falling into each bin on the y-axis. A two-sample Kolmogorov-Smirnov test was applied to each pair of data to test whether the function of correlations between RNAPII occupancies and histone modifications was less or greater than the function of similar correlations after one time point shift forward or backward in RNAPII profile. The statistical significance (p) of the correlation after the shift can be seen at the bottom of each graph. Control: correlations between RNAPII binding and histone modification within a single time point (e.g. T2). Shift HM -8 h: Comparison of RNAPII binding to histone modification (HM) in place 8 hours earlier. Shift HM +8 h: Comparison of RNAPII binding to histone modification (HM) in place 8 hours later. (TIFF 2 MB)

Additional file 7: Figure S6: Distribution of correlation between the binding of the Ser5-P form of RNAPII and the occupancy profiles of 13 histone modifications assessed before during and after RNAPII binding. Distribution of correlation between the binding of the Ser5-P form of RNAPII and the occupancy profiles of 13 histone modifications assessed at 8 h earlier (-8 h) or later (+8 h) than RNAPII binding. PCC was calculated between RNAPII profiles for all the probes with p <0.05 and ≥1.5 fold change and the corresponding histone mark after one-time point shift. For example, RNAPII binding at T2 was correlated to histone marks in place at T1 (-8 h) and T3 (+8 h). **(A)** Correlations with histone H3 modifications. **(B)** Correlations with histone H4 modifications. Correlation distributions were plotted as histograms for each histone modification, with bins of PCC ranging from -1 to +1 on x-axis plotted against the total number of probes falling into each bin on the y-axis. A two-sample Kolmogorov-Smirnov test was applied to each pair of data to test whether the function of correlations between RNAPII occupancies and histone modifications was less or greater than the function of similar correlations after one time point shift forward or backward in RNAPII profile. The statistical significance (p) of the correlation after the shift can be seen at the bottom of each graph. Control: correlations between RNAPII binding and histone modification within a single time point (e.g. T2). Shift HM -8 h: Comparison of RNAPII binding to histone modification (HM) in place 8 hours earlier. Shift HM +8 h: Comparison of RNAPII binding to histone modification (HM) in place 8 hours later. (TIFF 1 MB)

Additional file 8: Figure S7: Distribution of correlation between the binding of the Ser2/5-P form of RNAPII and the occupancy profiles of 13 histone modifications assessed before during and after RNAPII binding. Distribution of correlation between the binding of the Ser2/5-P form of RNAPII and the occupancy profiles of 13 histone modifications assessed at 8 h earlier (-8 h) or later (+8 h) than RNAPII binding. PCC was calculated between RNAPII profiles for all the probes with p <0.05 and ≥1.5 fold change and the corresponding histone mark after one-time point shift. For example, RNAPII binding at T2 was correlated to histone marks in place at T1 (-8 h) and T3 (+8 h). **(A)** Correlations with histone H3 modifications. **(B)** Correlations with histone H4 modifications. Correlation distributions were plotted as histograms for each histone modification, with bins of PCC ranging from -1 to +1 on x-axis plotted against the total number of probes falling into each bin on the y-axis. A two-sample Kolmogorov-Smirnov test was applied to each pair of data to test whether the function of correlations between RNAPII occupancies and histone modifications was less or greater than the function of similar correlations after one time point shift forward or backward in RNAPII profile. The statistical significance (p) of the correlation after the shift can be seen at the bottom of each graph. Control: correlations between RNAPII binding and histone modification within a single time point (e.g. T2). Shift HM -8 h: Comparison of RNAPII binding to histone modification (HM) in place 8 hours earlier. Shift HM +8 h: Comparison of RNAPII binding to histone modification (HM) in place 8 hours later. (TIFF 1 MB)

Additional file 9: Table S2: Oligonucleotides used in this study. (DOCX 25 KB)

## Abbreviations

ac: Acetyl
bp: Base pair (s)
ChIP: Chromatin immunoprecipitation
CTD: C-terminal domain
GO: Gene Ontology
H3: Histone 3
H4: Histone 4
hpi: Hours post-infection
IDC: Intraerythrocytic developmental cycle
kb: Kilo-base-pair (s)
KEGG: Kyoto Encyclopedia of Genes and Genomes
KNN: Kth nearest neighbor
me: Methyl
MPMP: Malaria Parasite Metabolic Pathway
PCC: Pearson’s Correlation Coefficient
RNAPII: RNA polymerase II
T1: T2…, time point 1, time point 2…
TAF: TBP-associated factor
TAP: Transcription activating proteins
TBP: TATA-binding protein
TFIID: Transcription factor II D
TFIIE: Transcription factor II E.
