# Supplementary figures and images for: Genome-wide analysis in Plasmodium falciparum reveals early and late phases of RNA polymerase II occupancy during the infectious cycle
Source: BMC Genomics. 2014 Nov 6;15(1):959. doi: 10.1186/1471-2164-15-959 (PMC4232647; doi:10.1186/1471-2164-15-959)

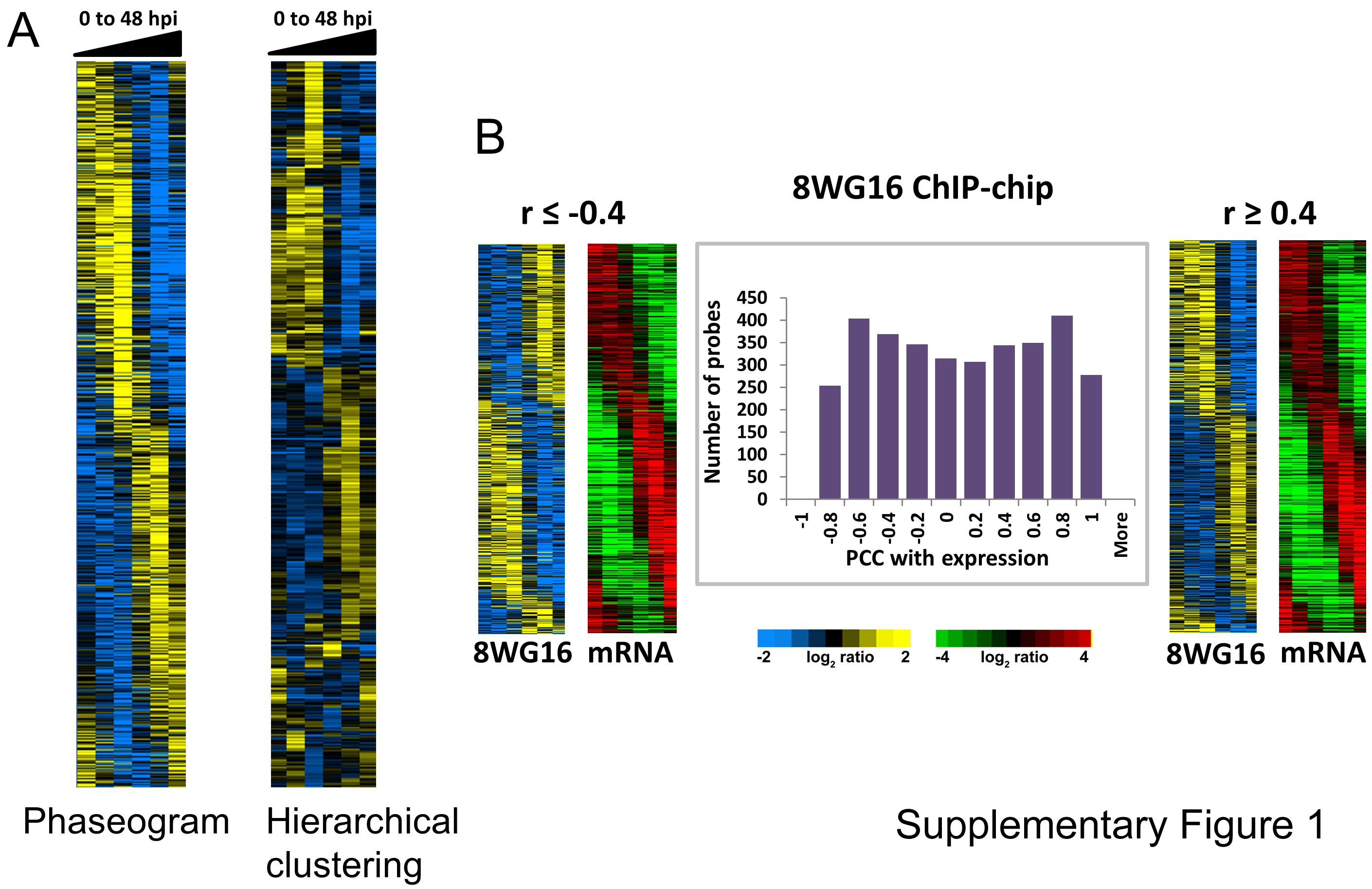

Supplement: Supplementary file 2 — Additional file 2: Figure S1: Results of ChIP-chip analysis using the commercial anti-CTD monoclonal antibody 8WG16. ChIP-chip was performed in duplicate and only probes with signals showing a fold change ≥1.5 across the life cycle in both experiments were included for analysis. (A) Heat maps showing phaseogram and hierarchical clustering of loci bound by RNAPII. (B) A comparable analysis to those presented in Figure 3. Approximately one third of probes bound by RNAPII are strongly positively correlated with mRNA levels (r ≥0.4) while a distinct third is strongly negatively correlated (r ≤ -0.4). (TIFF 818 KB) [file 12864_2014_6646_MOESM2_ESM.tiff]

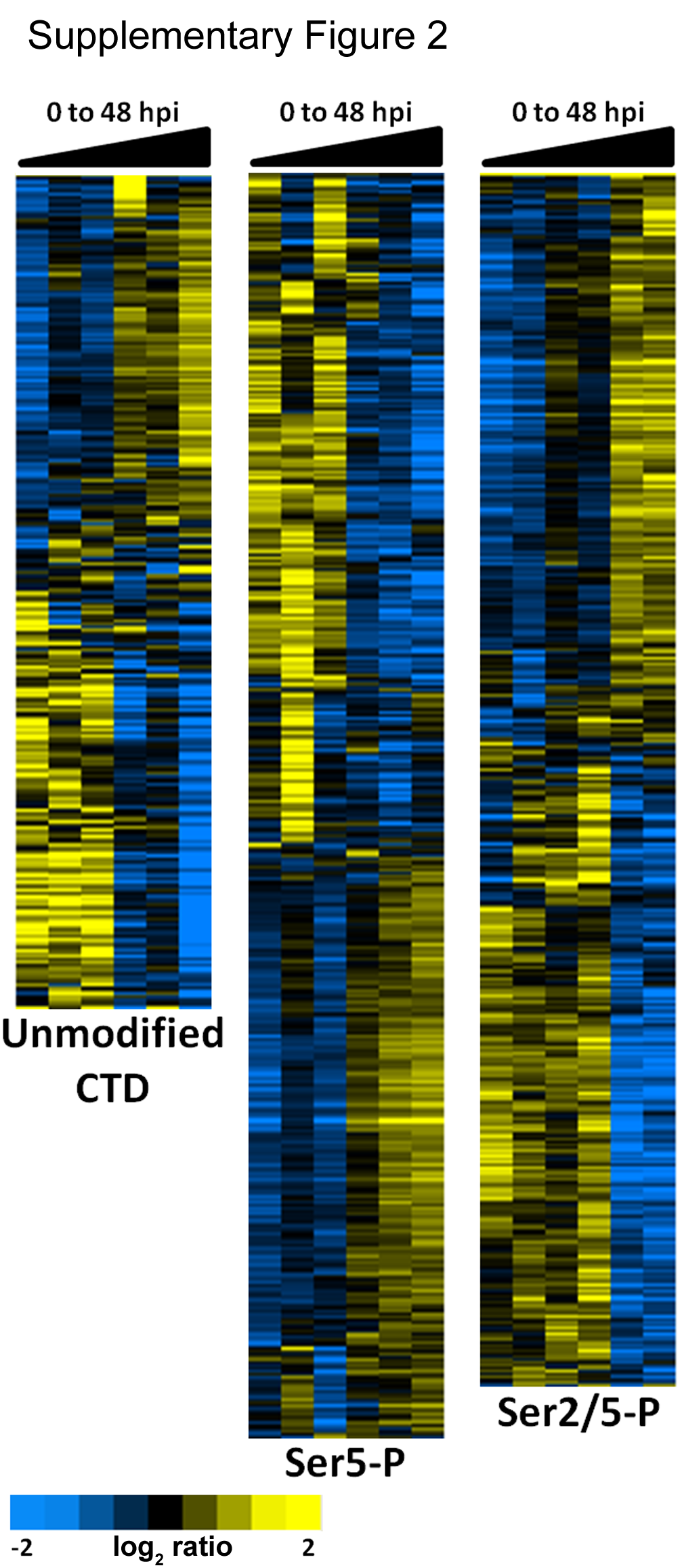

Supplement: Supplementary file 3 — Additional file 3: Figure S2: A hierarchical clustering of the microarray data presented in Figure 2A. A hierarchical clustering of log-transformed ChIP/input ratio of RNAPII occupancy for those loci where RNAPII shows an oscillating profile with p <0.05 and fold change ≥1.5 across the life cycle. (TIFF 892 KB) [file 12864_2014_6646_MOESM3_ESM.tiff]

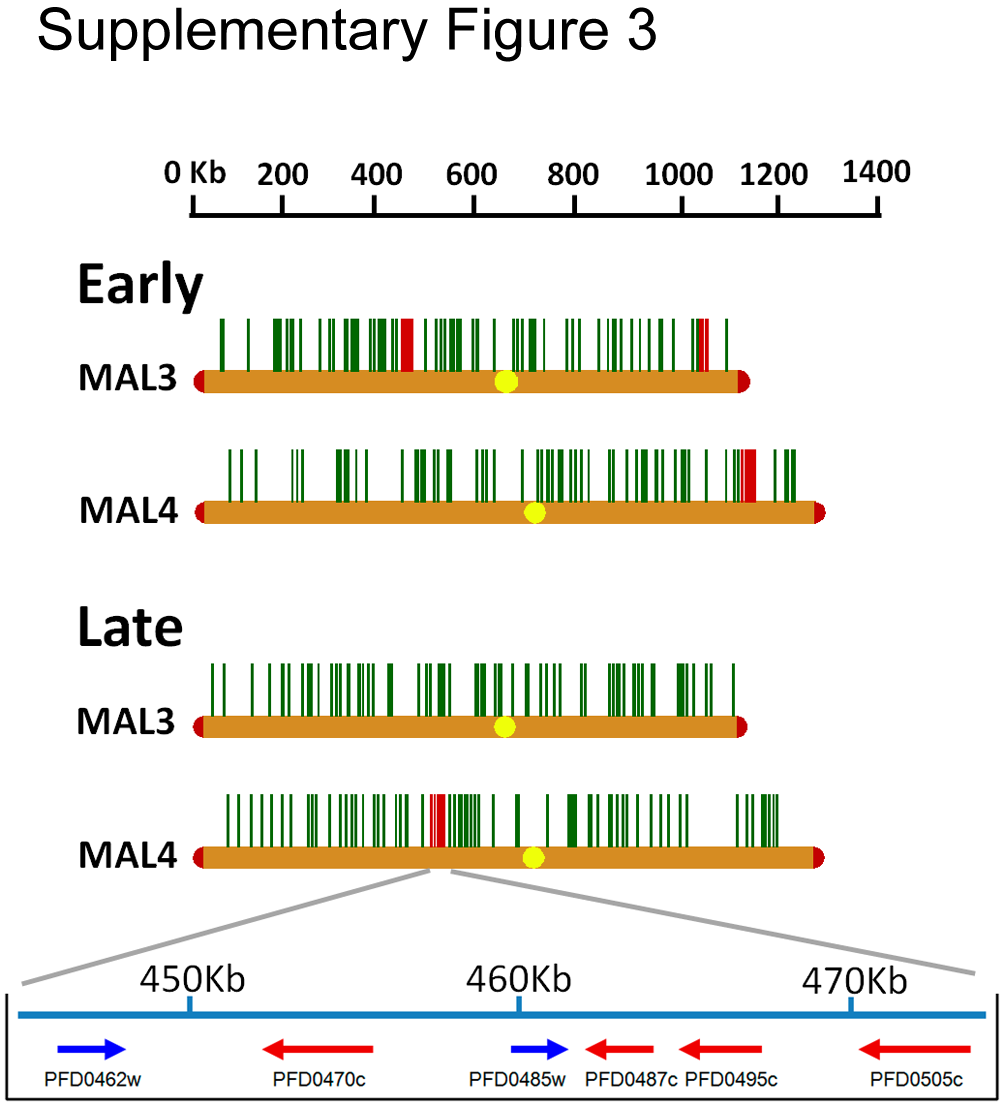

Supplement: Supplementary file 4 — Additional file 4: Figure S3: A representative example of the chromosomal distribution of genes identified in this study that bind RNAPII either early or late in the infectious cycle. Chromosomal projection of RNAPII Ser2/5-P occupancy for early and late gene groups onto chromosomes 3 (MAL3) and 4 (MAL4). The vertical bars (green) indicate probe position for each gene group. Gene clusters can be seen in red, the results for a “late” cluster on chromosome 4 is shown. Vertical bars denote the positions of late genes, and the bars in red indicate a cluster of 6 such genes spanning a mere 30 kb (shown to scale and with gene names and their transcriptional orientation in the bottom half of the figure). (TIFF 242 KB) [file 12864_2014_6646_MOESM4_ESM.tiff]

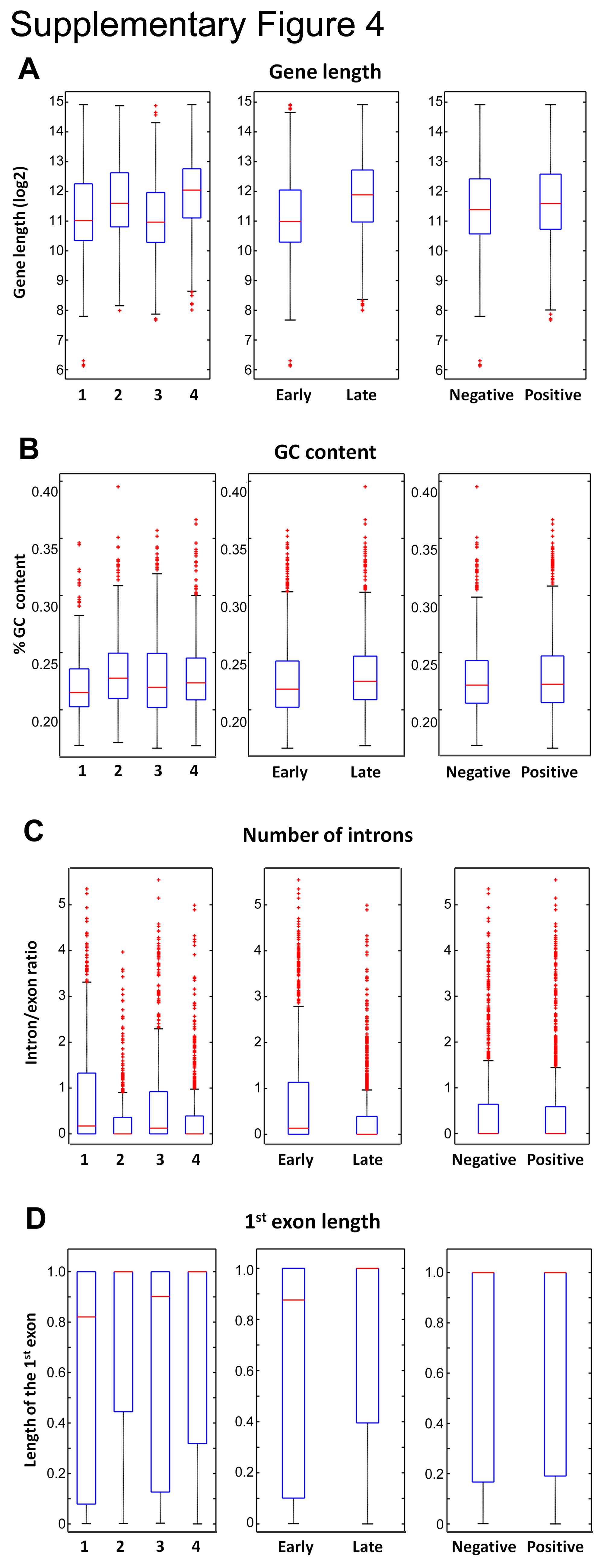

Supplement: Supplementary file 5 — Additional file 5: Figure S4: An analysis of the genomic features (gene length, GC content, number of introns, first exon length) in the four classes of genes identified in this study. Distinct genomic properties of early vs late genes. Box plots show the distribution of (A) gene length, (B) percentage GC content, (C) intron length (intron/exon ratio) and (D) length of the first exon for the early and late genes. 1, early/negative, 2, late/negative, 3, early/positive and 4, late/positive. p value for the two group comparisons is calculated using Wilcoxon two-tailed test and the p-value for the four group comparison is calculated using “Kruskal-Wallis test”. (TIFF 1 MB) [file 12864_2014_6646_MOESM5_ESM.tiff]

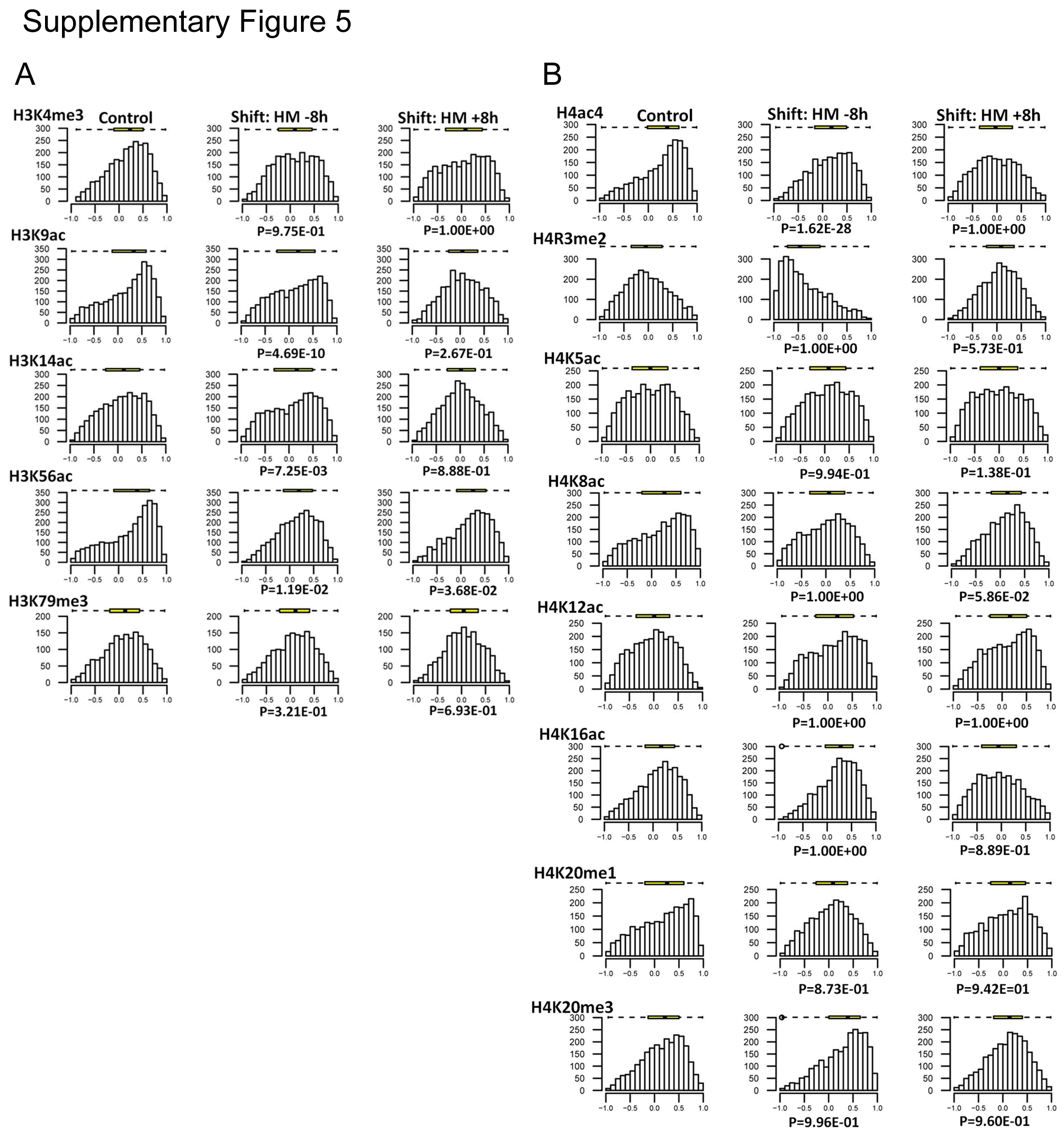

Supplement: Supplementary file 6 — Additional file 6: Figure S5: Distribution of correlation between the binding of the unmodified-CTD form of RNAPII and the occupancy profiles of 13 histone modifications assessed before during and after RNAPII binding. Distribution of correlation between the binding of the unmodified-CTD form of RNAPII and the occupancy profiles of 13 histone modifications assessed at 8 h earlier (-8 h) or later (+8 h) than RNAPII binding. PCC was calculated between RNAPII profiles for all the probes with p <0.05 and ≥1.5 fold change and the corresponding histone mark after one-time point shift. For example, RNAPII binding at T2 was correlated to histone marks in place at T1 (-8 h) and T3 (+8 h). (A) Correlations with histone H3 modifications. (B) Correlations with histone H4 modifications. Correlation distributions were plotted as histograms for each histone modification, with bins of PCC ranging from -1 to +1 on x-axis plotted against the total number of probes falling into each bin on the y-axis. A two-sample Kolmogorov-Smirnov test was applied to each pair of data to test whether the function of correlations between RNAPII occupancies and histone modifications was less or greater than the function of similar correlations after one time point shift forward or backward in RNAPII profile. The statistical significance (p) of the correlation after the shift can be seen at the bottom of each graph. Control: correlations between RNAPII binding and histone modification within a single time point (e.g. T2). Shift HM -8 h: Comparison of RNAPII binding to histone modification (HM) in place 8 hours earlier. Shift HM +8 h: Comparison of RNAPII binding to histone modification (HM) in place 8 hours later. (TIFF 2 MB) [file 12864_2014_6646_MOESM6_ESM.tiff]

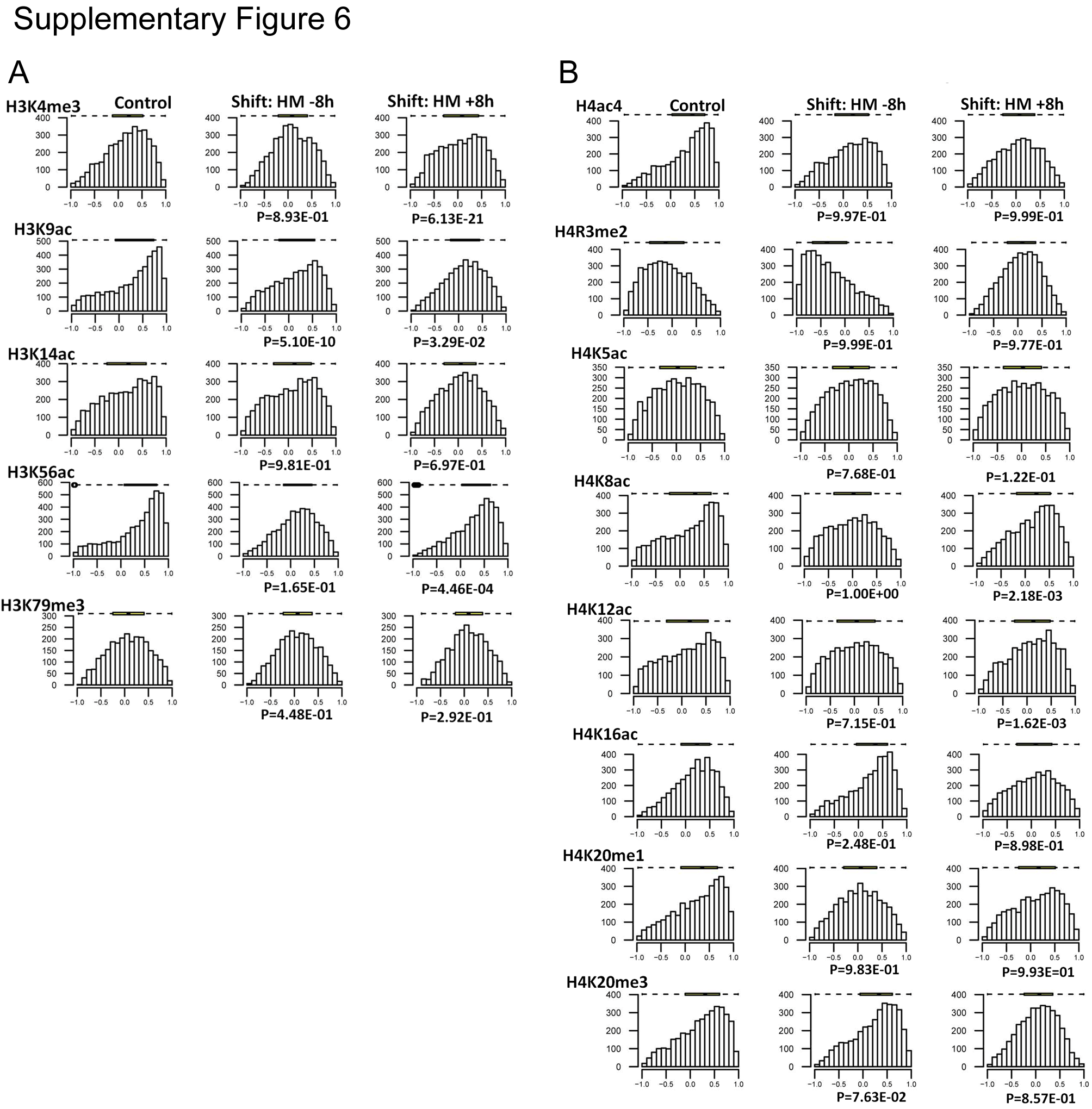

Supplement: Supplementary file 7 — Additional file 7: Figure S6: Distribution of correlation between the binding of the Ser5-P form of RNAPII and the occupancy profiles of 13 histone modifications assessed before during and after RNAPII binding. Distribution of correlation between the binding of the Ser5-P form of RNAPII and the occupancy profiles of 13 histone modifications assessed at 8 h earlier (-8 h) or later (+8 h) than RNAPII binding. PCC was calculated between RNAPII profiles for all the probes with p <0.05 and ≥1.5 fold change and the corresponding histone mark after one-time point shift. For example, RNAPII binding at T2 was correlated to histone marks in place at T1 (-8 h) and T3 (+8 h). (A) Correlations with histone H3 modifications. (B) Correlations with histone H4 modifications. Correlation distributions were plotted as histograms for each histone modification, with bins of PCC ranging from -1 to +1 on x-axis plotted against the total number of probes falling into each bin on the y-axis. A two-sample Kolmogorov-Smirnov test was applied to each pair of data to test whether the function of correlations between RNAPII occupancies and histone modifications was less or greater than the function of similar correlations after one time point shift forward or backward in RNAPII profile. The statistical significance (p) of the correlation after the shift can be seen at the bottom of each graph. Control: correlations between RNAPII binding and histone modification within a single time point (e.g. T2). Shift HM -8 h: Comparison of RNAPII binding to histone modification (HM) in place 8 hours earlier. Shift HM +8 h: Comparison of RNAPII binding to histone modification (HM) in place 8 hours later. (TIFF 1 MB) [file 12864_2014_6646_MOESM7_ESM.tiff]

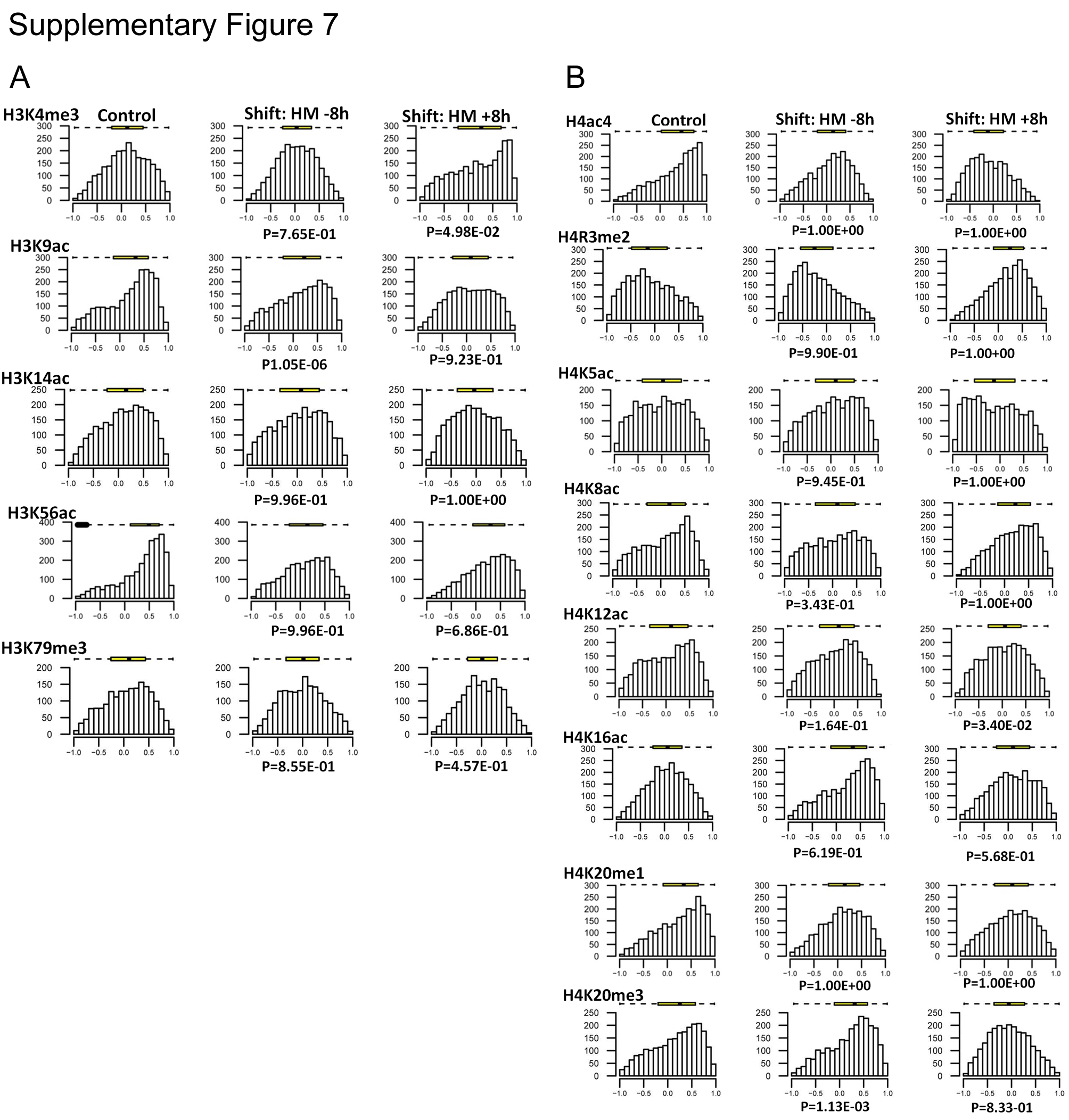

Supplement: Supplementary file 8 — Additional file 8: Figure S7: Distribution of correlation between the binding of the Ser2/5-P form of RNAPII and the occupancy profiles of 13 histone modifications assessed before during and after RNAPII binding. Distribution of correlation between the binding of the Ser2/5-P form of RNAPII and the occupancy profiles of 13 histone modifications assessed at 8 h earlier (-8 h) or later (+8 h) than RNAPII binding. PCC was calculated between RNAPII profiles for all the probes with p <0.05 and ≥1.5 fold change and the corresponding histone mark after one-time point shift. For example, RNAPII binding at T2 was correlated to histone marks in place at T1 (-8 h) and T3 (+8 h). (A) Correlations with histone H3 modifications. (B) Correlations with histone H4 modifications. Correlation distributions were plotted as histograms for each histone modification, with bins of PCC ranging from -1 to +1 on x-axis plotted against the total number of probes falling into each bin on the y-axis. A two-sample Kolmogorov-Smirnov test was applied to each pair of data to test whether the function of correlations between RNAPII occupancies and histone modifications was less or greater than the function of similar correlations after one time point shift forward or backward in RNAPII profile. The statistical significance (p) of the correlation after the shift can be seen at the bottom of each graph. Control: correlations between RNAPII binding and histone modification within a single time point (e.g. T2). Shift HM -8 h: Comparison of RNAPII binding to histone modification (HM) in place 8 hours earlier. Shift HM +8 h: Comparison of RNAPII binding to histone modification (HM) in place 8 hours later. (TIFF 1 MB) [file 12864_2014_6646_MOESM8_ESM.tiff]
