# Supplementary material for: Genome-wide analysis in Plasmodium falciparum reveals early and late phases of RNA polymerase II occupancy during the infectious cycle
Source: BMC Genomics. 2014 Nov 6;15(1):959. doi: 10.1186/1471-2164-15-959 (PMC4232647; doi:10.1186/1471-2164-15-959)
Supplement: Supplementary file 9 — Additional file 9: Table S2: Oligonucleotides used in this study. (DOCX 25 KB) [file 12864_2014_6646_MOESM9_ESM.docx]

**Additional file 9: Table S2 Oligonucleotides used in this study**

| **s/no.** | **Category** | **Primers** | **Sequence** | **Target gene** |
| --- | --- | --- | --- | --- |
| 1 | ChIP-chip | Random A | GTTTCCCAGTCACGATCNNNNNNNNN |  |
| 2 | ChIP-chip | Random B | GTTTCCCAGTCACGATC |  |
| 3 | ChIP-qPCR | aMAL13P1.294_1_F1 | CGTTGAAGCTGTTCTTAACCAAAT | PF3D7_1358900 |
| 4 | ChIP-qPCR | aMAL13P1.294_1_R1 | CCTCATCAGCTAGTTTGTGATCTG |  |
| 5 | ChIP-qPCR | aPFL1830w_0_F1 | AAAGAATAAACTTGGAGAGTGTGC | PF3D7_1237800 |
| 6 | ChIP-qPCR | aPFL1830w_0_R1 | TCGTATTTTATCTGCTCTTGTACC |  |
| 7 | ChIP-qPCR | aPFL1410c_3_F1 | TACCTTTTCATGTAGCCTTGGT | PF3D7_1229100 |
| 8 | ChIP-qPCR | aPFL1410c_3_R1 | CATACGAACCAGGAACAGATTT |  |
| 9 | ChIP-qPCR | aMAL13P1.28_0_F1 | CAAGAAAAGGTAGATATGTTAAAAGG | PF3D7_1305400 |
| 10 | ChIP-qPCR | aMAL13P1.28_0_R1 | AATTCGAATAATCAAAACCTATGTG |  |
| 11 | ChIP-qPCR | aPF10_0270_0_F1 | CAATAACAGCAGGAAAGTGTTGTA | PF3D7_1027500 |
| 12 | ChIP-qPCR | aPF10_0270_0_R1 | GTCGTTCTTCAACCTTCTCTTATC |  |
| 13 | ChIP-qPCR | aPF10_0114_0_F1 | AACACTACAAAACAACGAAGAGG | PF3D7_1011700 |
| 14 | ChIP-qPCR | aPF10_0114_0_R1 | CTGTTGCTTTGTCTTCATCTTTTA |  |
| 15 | ChIP-qPCR | aPFL2215w_0 _F1 | GGAAATCGTGAGAGGATGACA | PF3D7_1246200 |
| 16 | ChIP-qPCR | aPFL2215w_0 _R1 | TCTGCTGATGTTGAAAATCCAT |  |
| 17 | ChIP-qPCR | aPFI1475w_1_F1 | GCAAAATTAAATGATGTATGTGCT | PF3D7_0930300 |
| 18 | ChIP-qPCR | aPFI1475w_1_R1 | TCCCTTCTTCATTATCTGCATTC |  |
| 19 | ChIP-qPCR | aPFA0125c_2_F1 | GCATGCGACAGATAATTCTGA | PF3D7_0102500 |
| 20 | ChIP-qPCR | aPFA0125c_2_R1 | TGTTTCCCCCTTCGTCTCTAT |  |
| 21 | ChIP-qPCR | aPF10_0346_1_F1 | CAGAAACAGAAAATTTGGAAACA | PF3D7_1035500 |
| 22 | ChIP-qPCR | aPF10_0346_1_R1 | TTTCATTAAATAATCCAACCAATG |  |
